# Gap junctions in Turing-type periodic feather pattern formation

**DOI:** 10.1371/journal.pbio.3002636

**Published:** 2024-05-14

**Authors:** Chun-Chih Tseng, Thomas E. Woolley, Ting-Xin Jiang, Ping Wu, Philip K. Maini, Randall B. Widelitz, Cheng-Ming Chuong

**Affiliations:** 1 Department of Biochemistry and Molecular Medicine, USC Norris Comprehensive Cancer Center, Keck School of Medicine, University of Southern California, Los Angeles, California, United States of America; 2 School of Mathematics, Cardiff University, Cardiff, United Kingdom; 3 Department of Pathology, University of Southern California, Los Angeles, California, United States of America; 4 Wolfson Centre for Mathematical Biology, Mathematical Institute, Andrew Wiles Building, University of Oxford, Radcliffe Observatory Quarter, Oxford, United Kingdom; HudsonAlpha Institute for Biotechnology, UNITED STATES

## Abstract

Periodic patterning requires coordinated cell–cell interactions at the tissue level. Turing showed, using mathematical modeling, how spatial patterns could arise from the reactions of a diffusive activator-inhibitor pair in an initially homogeneous 2D field. Most activators and inhibitors studied in biological systems are proteins, and the roles of cell–cell interaction, ions, bioelectricity, etc. are only now being identified. Gap junctions (GJs) mediate direct exchanges of ions or small molecules between cells, enabling rapid long-distance communications in a cell collective. They are therefore good candidates for propagating nonprotein-based patterning signals that may act according to the Turing principles. Here, we explore the possible roles of GJs in Turing-type patterning using feather pattern formation as a model. We found 7 of the 12 investigated GJ isoforms are highly dynamically expressed in the developing chicken skin. In ovo functional perturbations of the GJ isoform, *connexin 30*, by siRNA and the dominant-negative mutant applied before placode development led to disrupted primary feather bud formation. Interestingly, inhibition of gap junctional intercellular communication (GJIC) in the ex vivo skin explant culture allowed the sequential emergence of new feather buds at specific spatial locations relative to the existing primary buds. The results suggest that GJIC may facilitate the propagation of long-distance inhibitory signals. Thus, inhibition of GJs may stimulate Turing-type periodic feather pattern formation during chick skin development, and the removal of GJ activity would enable the emergence of new feather buds if the local environment were competent and the threshold to form buds was reached. We further propose Turing-based computational simulations that can predict the sequential appearance of these ectopic buds. Our models demonstrate how a Turing activator-inhibitor system can continue to generate patterns in the competent morphogenetic field when the level of intercellular communication at the tissue scale is modulated.

## Abstract 2

Gap junctions mediate direct exchanges of ions or small molecules between cells, enabling rapid long-distance communication. This study shows that inhibition of gap junction intercellular communication can stimulate new feather bud formation in specific topological positions in developing chick skin explants, which can be predicted with mathematical simulations of consecutive waves of Turing’s instabilities.

## Introduction

**Fig 1:**
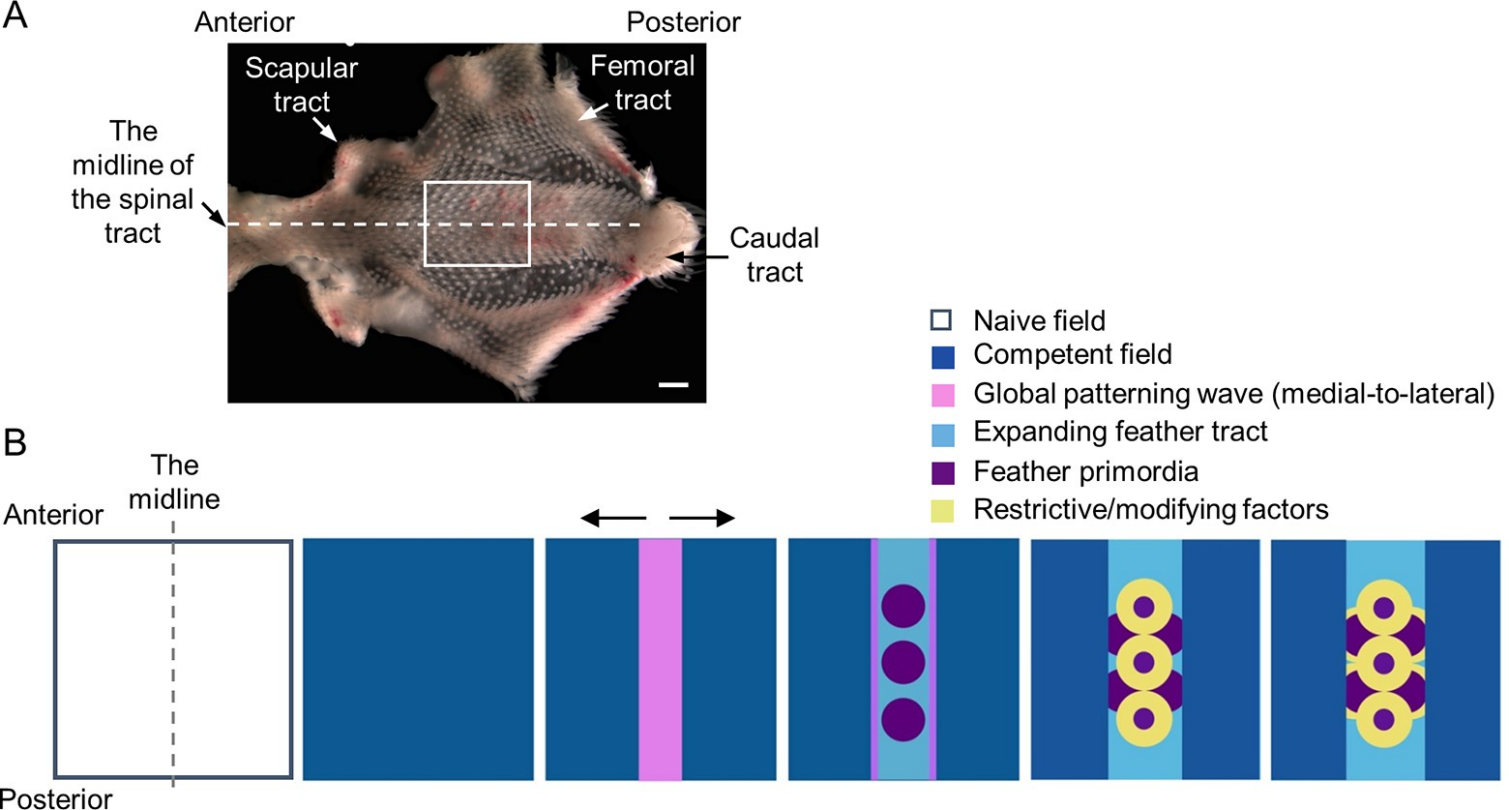
Schematic illustrating the emergence of chicken feather primordia. (A) The chick skin contains discrete feathered areas, termed tracts. In H&H stage 40 chick dorsal skin, we highlight 4 of the tracts: the spinal tract, the scapular tract, the femoral tract, and the caudal tract. Anterior of the embryo is toward the left. Scale bar, 1,000 μm. Developmental changes that occur within the boxed region of the thoracic-lumbar region are schematized below. (B) Feather formation starts from a field lacking any evident patterning cues, followed by the emergence of competent patterning fields. In the spinal tract, the highly ordered feather array is formed along the midline and then progressively and bilaterally expanded across the skin. While the patterning wave(s) travel through the skin, the spatiotemporal pattern and the size of feather primordia are sequentially established by promoting and restricting/modifying factors. The triangle-headed arrows show the directions of the patterning waves. Anterior is to the upper side.

In 1952, Alan Turing published his seminal paper describing how spatial patterns can arise from 2 reactive and diffusive factors [1], termed morphogens, in a system which exhibits spatially uniform steady states that are stable in the absence of diffusion. However, 72 years later, we still have a limited understanding of the identity of the morphogens and how these morphogens can propagate through complex biological systems [2,3]. Here, we use the developing chicken skin to further our understanding. During embryonic development, feathers form in discrete regions over the body surface, called tracts or pterylae, that are distributed across the avian skin (Fig 1A) [4]. Featherless regions lying between tracts are referred to as apteric regions. Chicken skin exhibits characteristic hexagonal feather arrays, which are best demonstrated in the spinal tract. In the spinal tract, the skin forms a competent feather field and then the first row of feather primordia appears along the midline. Subsequent rows of feathers spread bilaterally, adding more rows of younger feather primordia (Fig 1B) [5,6]. These have been documented to form due to chemical reactions and mechanical forces that trigger the self-organization of mesodermal cells. The resultant mesodermal cell aggregates then promote the epidermal expression and nucleation of a key regulator of feather morphogenesis, β–catenin [7,8]. The periodicity of the feather primordia is at least in part ensured by a global ectodysplasin A (EDA) patterning wave expanding from the midline of the spinal tract (Fig 1B), which lowers the mesenchymal cell density threshold needed to form feather primordia [9,10]. Locally, Turing’s theory of diffusion-driven instability describes how a pair of reaction–diffusion (RD) equations with activator-inhibitor kinetics and randomly perturbed initial conditions in a homogeneous field can lead to periodic pattern formation [1]. Based on this model, a self-amplifying short-range activator can stimulate the production of a long-range inhibitor that, in turn, negatively regulates the activator. Previous studies showed that a variety of spatial patterns can be generated in simulated mathematical RD model systems [3,11,12]. In addition, a global patterning wave involving mechanochemical reactions allows the dermal cells to aggregate [9,13,14] and together they produce a highly ordered hexagonal patterned array of feather primordia in the chicken. On the other hand, without the global wave, local Turing patterning can produce periodic buds simultaneously in the tract [9,14], similar to the patterning process in reconstituted feather explants [7].

Importantly, Turing’s RD theory can be applied to a diverse range of integuments, such as the FGF/SHH-BMP pair in periodic feather patterning, shark skin denticle patterning [9,15–17], and the WNT-DKK pair in hair follicle spacing [18]. Most of the morphogens discovered in animals are secreted proteins which diffuse. For example, *Pax6* establishes a *Tgfb2* and *Fst* Turing signaling network that has been proposed to pattern chick eye cup development [19]. The role of FGF, hedgehog, Wnt, and BMP signaling in establishing the murine periodic striped patterning of rugae ridges on the oral palate was identified using inhibitors [20]. The roles of ions, bioelectricity, small molecules, and cell–cell contacts in periodic patterning are just emerging. Notably, cell–cell interactions between pigment cells are required for zebrafish stripe and spot patterning [21]. Although the underlying molecular mechanism of zebrafish pigment pattern formation is still elusive, it is, at least in part, regulated by spermine, a small poly-cation, and its regulation of the flow of plasma membrane channels, including the potassium channel, Kir7.1, and gap junctions, Cx41.8 and Cx39.4 [21–26].

Gap junctions (GJs) are formed by direct docking of connexon or pannexon hemichannels consisting of 6 subunits of connexin (Cx) or pannexin (Panx) family proteins, respectively, between adjacent cells. GJs allow the direct exchange of cellular content, including ions, small metabolites, and second messengers, between neighboring cells [27–29]. Connexins and pannexins form distinct families of GJ subunits. There are 21 connexins and 3 pannexins within the human genome and at least 12 connexins and 3 pannexins identified or predicted within the chick genome. The conserved domains, including N-terminus, extracellular loops, and transmembrane domains in connexin families, have shown great sequence homology across vertebrate species [30]. Pannexins share some sequence homology with invertebrate gap junctions, innexins, but show no sequence homology with connexins [31]. GJs formed by Cx or Panx possess distinct electrophysiological and pharmacological properties. Panx GJs are less sensitive to the change in trans-junctional voltage compared to Cx GJs, and Panx GJs favor anionic substrates over cations [32]. Many of these GJ isoforms exhibit tissue-specific and overlapping expression patterns. The “knock in” experiment, a genetic technique that replaces a gene with another one in the genomic locus, performed in mouse showed that individual connexins may play distinct and shared roles in specific cellular processes [33]. In agreement with this, studies have found that the heterotypic and heteromeric types of connexins are able to form functional channels. GJs formed by different types of Cxs exhibited a selective permeability to cytoplasmic molecules [34]. Importantly, many studies have demonstrated that GJs not only mediate the intercellular exchange of hydrophilic molecules but also act as a regulatable signaling scaffold that can be finely tuned by posttranslational modifications, such as phosphorylation and ubiquitination and form complexes with a variety of junctional and signaling molecules [34–36].

Connexins and pannexins are expressed in diverse patterns in skin and skin appendages [37–40]. Transcriptome profiling has revealed that multiple connexin and pannexin isoforms are expressed during embryonic feather morphogenesis (H&H stages 31 and 35). Among them, *Cx43* is the most abundantly expressed isoform [38]. The same study examined the functions of Cx43 in greater detail and showed that Cx43 can facilitate the propagation of intercellular calcium signaling and regulate mesenchymal cell migration during elongation of feather buds [38]. In an earlier study, functional coupling of GJs between cells during early feather morphogenesis was demonstrated by the transfer of Lucifer yellow (LY) injected into a single cell, where the gap junctional intercellular communication (GJIC) exhibits asymmetric diffusion and differential compartmentalization between bud and interbud regions in the chick skin (H&H stage 30 to 35) [41].

Periodic feather pattern formation requires coordinated cell–cell interactions. The properties of GJs that allow them to facilitate the exchange of cellular information between adjacent cells and act as a signaling platform at cell–cell junctions make the GJs a viable candidate to be involved in feather patterning. The previous studies mentioned above have shown that GJIC is a highly dynamic process and is associated with compartmentalization during feather morphogenesis. GJIC and the specific GJ isoform, Cx43, are important for the elongation of individual feather buds. However, these studies only addressed limited aspects of the functional importance of GJIC during feather morphogenesis, and the roles of GJ isoforms in periodic patterning are still largely unknown. In the present study, we focus on the connexin family and show that 7 of the 12 tested connexins are expressed during early feather morphogenesis (H&H stage 28 to 35). We found that *Cx30* and *Cx43* are the earliest detected connexin isoforms during feather patterning as demonstrated by whole-mount in situ hybridization. Functional perturbations of Cx30 by introducing siRNA and RCAS virus carrying a COOH-terminal truncated Cx30 mutant (a.a. 1–214) resulted in suppression of feather bud formation. Inhibition of GJIC activities by small molecule GJ channel blockers, 18 α-glycyrrhetinic acid (AGA) and its derivative, in ex vivo cultured chicken skin explants allows the emergence of ectopic feather buds through consecutive Turing instabilities or in specific locally competent tissues. AGA treatment activates the expression of *β-catenin* and *Shh*, early markers of feather morphogenesis, and stimulates cell proliferation in the ectopic buds. These results suggest that diffusing factors passing through gap junctions during normal feather patterning may serve as the long-range inhibitory signals described in Turing’s model. Overall, this study uncovers unexpected roles of GJs in feather patterning and provides new insights into how long-range direct diffusion through GJs can be coupled with Turing’s mechanism and local tissue competence, referred to the ability of tissues to sense and respond to environmental stimuli, to modulate diverse patterning outcomes at different morphogenetic stages.

## Results

### Connexins exhibit dynamic expression patterns during early feather morphogenesis

We used the identified or predicted NCBI nucleotide coding sequence for 12 chicken connexin isoforms to examine their expression at early stages (H&H stage 28 to 35) of feather morphogenesis using whole-mount in situ hybridization (WM-ISH) and tissue sectioning of the stained embryos. To avoid cross-reactivity between connexin isoforms, we designed primer sets for each connexin ensuring that WM-ISH probe sequences shared less than 55% similarity (S1 Table).

**Fig 2:**
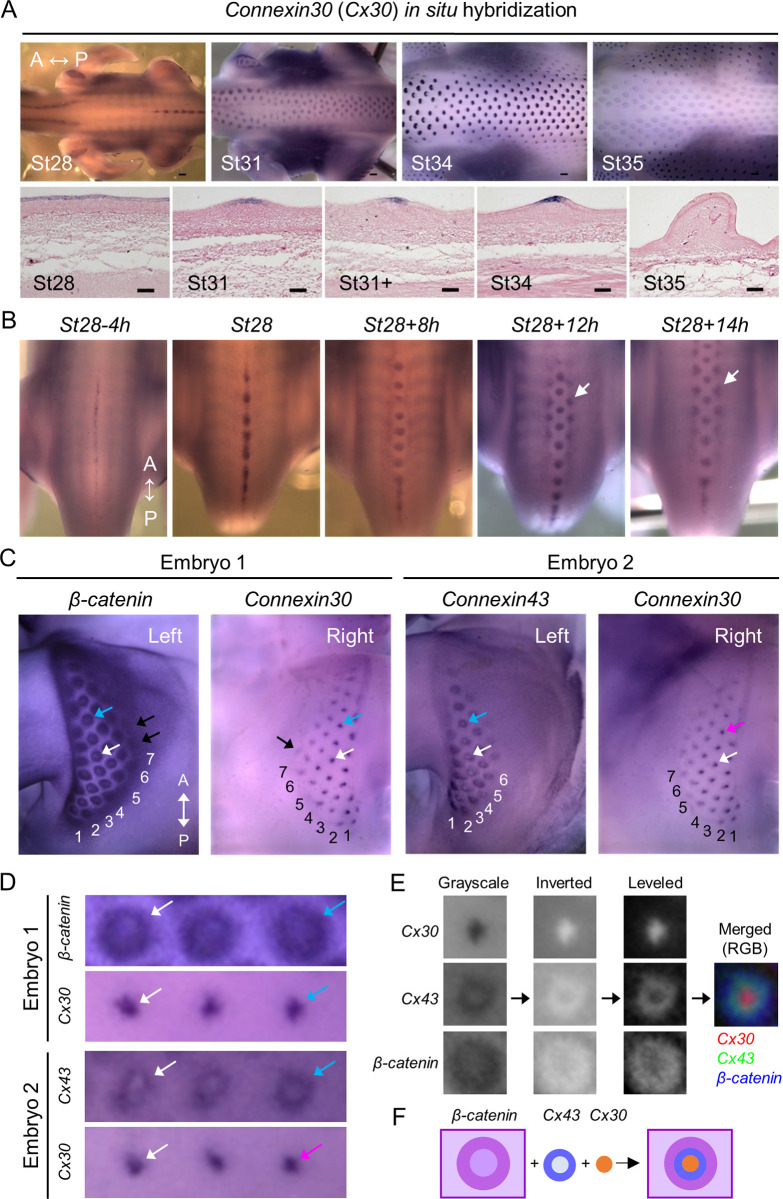
Dynamic expression of *connexin 30* (*Cx30*) during early feather morphogenesis. (A) *Cx30* expression in H&H stage (St) 28 to 35 chicken embryos. Upper panels: *Cx30* RNA was visualized by WM-ISH. Lower panels: After WM-ISH, the embryos were embedded in paraffin and then longitudinally sectioned along or near the midline of the spinal tract. Tissue sections were counter-stained with 10% eosin Y. Thickness of tissue sections: 14 μm. A, anterior. P, posterior. Scale bars, 300 μm (whole embryo); 50 μm (tissue sections). (B) *Cx30* RNA was visualized by WM-ISH in chicken embryos at or around H&H stage 28. Left to right: embryos were collected from earlier to later time points as indicated. The triangle-headed arrows indicate emerging feather primordia. (h, hours). A, anterior. P, posterior. (C) Gene expression of *β–catenin*, *Cx30*, or *Cx43* in the femoral tract was visualized by WM-ISH on contralateral sides of embryos at H&H stage 31. The numbers indicate feather primordium rows. The triangle-headed arrows in black indicate emerging feather primordia. A, anterior. P, posterior. The areas pointed out by the triangle-headed arrows in cyan, magenta, or white were enlarged in the corresponding images in (D). (E) The images of the feather primordia pointed out by the triangle-headed arrows in cyan in panel (D) were cropped and processed by Image J software with the indicated procedures to create an image with merged *Cx30*, *Cx43*, and *β–catenin* expressions. (F) Schematic summary of *Cx30*, *Cx43*, and *β–catenin* expressions in a feather primordium. WM-ISH, whole-mount in situ hybridization.

*Connexin 30* (*Cx30*) exhibited highly dynamic expression patterns at the pre-placode stage (H&H stage 28, St28) to the short bud stage (H&H stage 34) but was not detected at the long bud stage (H&H stage 35) (Fig 2A). At H&H stage 28, it emerged in the epithelium of the spinal tract as a midline stripe and then gradually became restricted to the bud epithelium at H&H stage 31 (Fig 2A and 2B). During feather primordia stabilization and polarization, *Cx30* expression in the bud became further restricted in size at the short bud stage anterior epithelium. The emerging *Cx30* transcripts were coupled with bilateral global traveling wave expansion, highlighted by arrows pointing to the partial circle/triangular-shaped in situ hybridization pattern (Fig 2B). The early *Cx30* expression prompted us to compare its expression with *β–catenin*, the feather tract and early placode marker, and *Cx43*, another GJ isoform that was previously identified by bulk RNA-seq as the most abundantly expressed GJ during early feather patterning [38]. The transitional bud morphogenesis process can be best observed in the femoral tract. By independently staining contralateral halves of embryos, we can compare the expression patterns of 2 different genes in the same embryo. As shown in Fig 2C, we observed the same number of rows of *β–catenin* and *Cx30* expression, but *β–catenin* showed expression in one additional primordium in row 7 compared to *Cx30* (black arrows). On the other hand, *Cx30* expression preceded *Cx43* expression by one additional row. Thus, we can conclude that among these 3 genes, *β–catenin* is detected earliest followed by *Cx30* and then *Cx43*. Interestingly, it appears that *Cx30* and *Cx43* expressions were mutually exclusive in the bud domain in more mature feather primordia. This can more easily be seen in the fourth feather row of embryo 2, where *Cx43* is expressed as a hollow circle while Cx30 is expressed as a central dot in feather primordia localized at comparable sites (Fig 2C, triangle-headed arrows in white, cyan, and magenta). A similar hollow *Cx43* pattern could also be observed in more mature feather primordia within the spinal tract (S1 Fig). A closer examination of the expression of *β–catenin* (Fig 2C, the leftmost panel) revealed a ring-shaped pattern similar to the hollow circle of the *Cx43* expression. To further characterize these observations (Fig 2D), we compared their expression patterns from the feather primordia located in comparable anatomical locations shown in Fig 2C (triangle-headed arrows in white, blue, or magenta). We observed that *Cx30* was expressed near the center of circles formed by *β–catenin* and *Cx43* expressions, and the circles formed by *β–catenin* expression seem bigger than the ones formed by *Cx43* expression. To better visualize the size differences of *Cx30*, *Cx43*, and *β–catenin* expressions in the feather primordia, we created a merged image from their expressions shown in the buds indicated by the triangle-headed arrows (in cyan) in Fig 2C and 2D in Image J software (Fig 2E). We use schematic drawings to summarize these observations in Fig 2F. The expressions of *Cx30*, *Cx43*, and *β–catenin* are likely concentric, thus raising an intriguing possibility of β–catenin in fine-tuning the compartmentation and/or maturation of feather primordia. It would also be of interest to further investigate how these proteins may collaborate with known activator/inhibitor signaling to regulate feather primordia formation.

**Fig 3:**
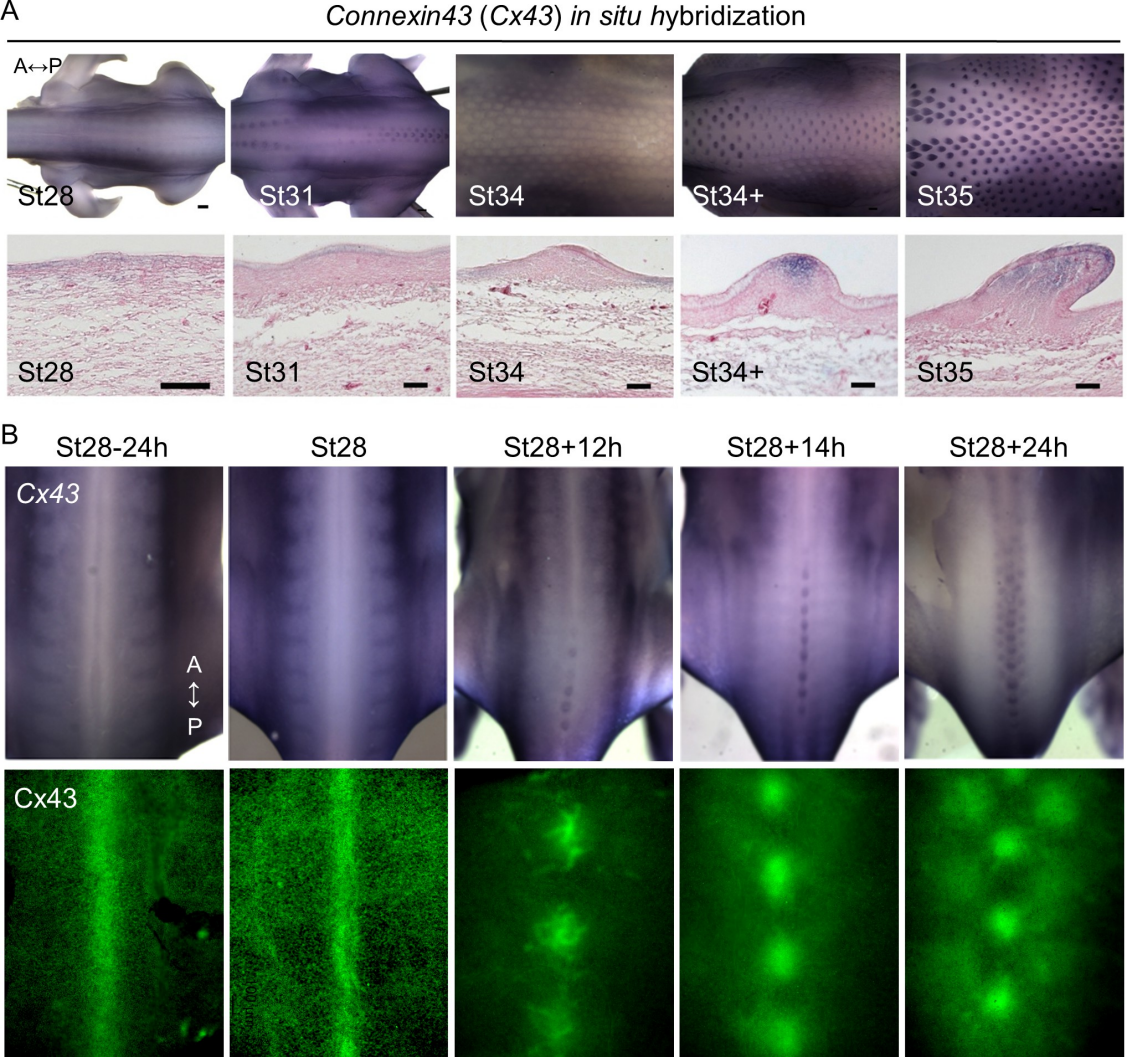
Expression of Cx43 during early feather morphogenesis. (A) Gene expression of *Cx43* in the chicken embryos between H&H stage 28 and 35. Upper panels: *Cx43* RNA was visualized by WM-ISH. Lower panels: After WM-ISH, the embryos were embedded in paraffin and then longitudinally sectioned along or near the midline of the spinal tract. Tissue sections were counter-stained with 10% eosin Y. Thickness of tissue sections: 14 μm. A, anterior. P, posterior. Scale bars, 300 μm (whole embryo); 50 μm (tissue sections). (B) Upper panels: *Cx43* RNA was visualized by WM-ISH in chicken embryos at, or around, H&H stage 28. Left to right: embryos were collected from earlier to later time points. (h, hours). Lower panels: protein expression of Cx43 in the dorsal skin of chicken embryos at the corresponding developmental stages as the upper panels. The embryos were stained with an antibody against Cx43 (Santa Cruz Biotechnology, sc-9059) by WM-immunostaining and then the dorsal skins were surgically removed for imaging. A, anterior. P, posterior. WM-ISH, whole-mount in situ hybridization.

*Connexin 43* showed highly dynamic expression patterns during feather patterning at H&H stage 28 to 35 as demonstrated by WM-ISH and tissue sectioning of the stained embryos (Fig 3A). At the pre-placode stage (H&H stage 28), *Cx43* is weakly expressed in both the mesoderm and the epithelium in the entire spinal tract. Since the resolution of WM-ISH could not clearly demonstrate whether *Cx43* expression is localized to the midline at St28 (Fig 3A and 3B, upper panels), we stained embryos with a chick Cx43 antibody (Santa Cruz Biotechnology, sc-9059) and showed that it was present in the entire spinal tract skin, exhibiting enhanced midline expression (Fig 3B, lower panels). Moreover, Cx43 protein was expressed not only at pre-placode stage (H&H stage28) but also at a much earlier stage (St28-24h). During placode formation (H&H stage 31), Cx43 expression was increased in the bud epithelium and undetectable in the mesoderm (Fig 3A and 3B). Interestingly, its expression swiftly became restricted to the interbud epithelium and mesoderm at H&H stage 34 when feather buds just started to show visible signs of asymmetric growth (Fig 3A). When the feather buds further elongate, *Cx43* expression appears in the distal epithelium and mesoderm of the bud domain (Fig 3A, St34+) and extends to the proximal region of the bud mesoderm (St35). *Cx43* expression is absent in the interbud at St34+ and St35.

**Fig 4:**
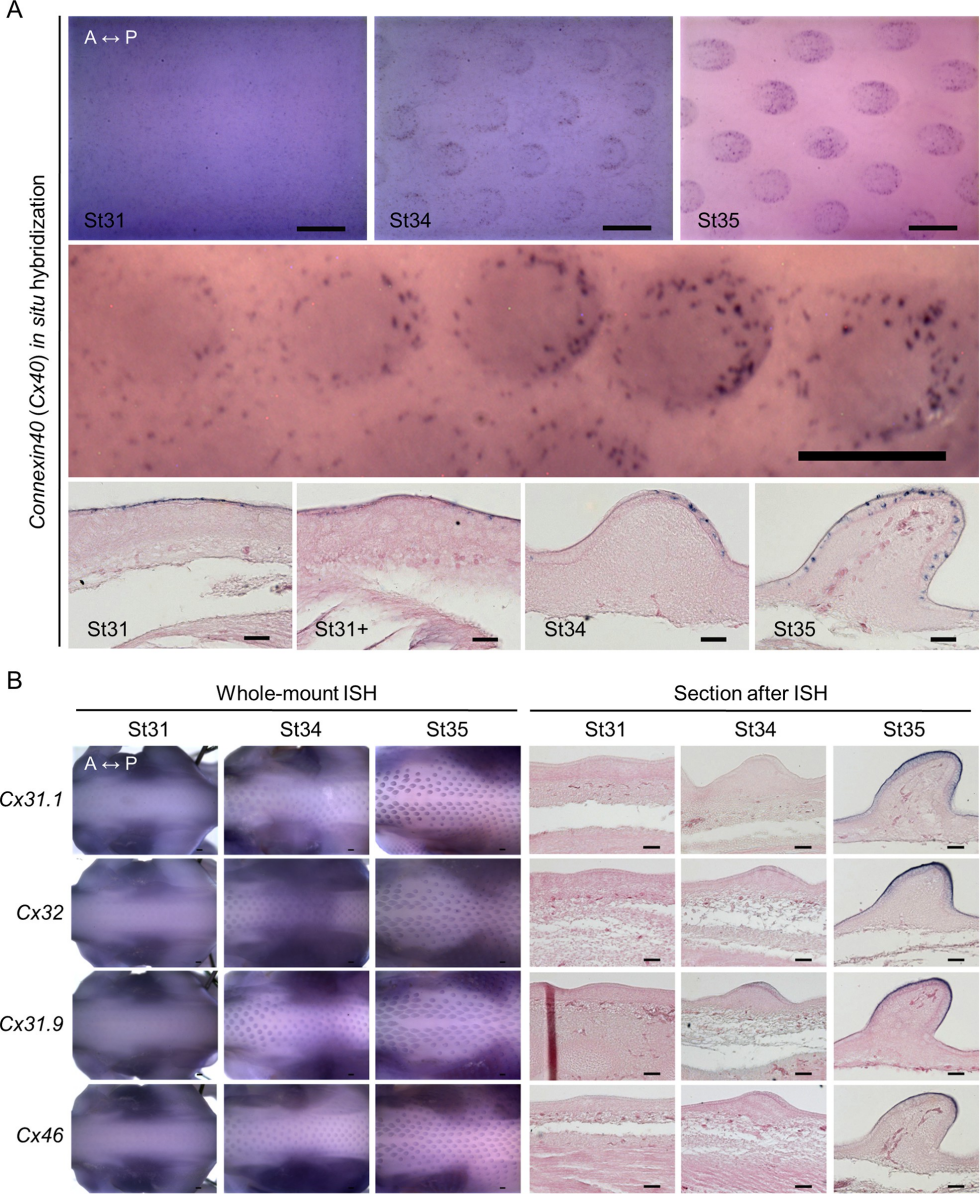
Expression of *Cx40*, *Cx31*.*1*, *Cx32*, *Cx31*.*9*, and *Cx46* during early feather morphogenesis. (A) Gene expression of *Cx40* in chicken embryos between H&H stage 31 and 35. Upper panels: dorsal views of embryonic thoracic-lumbar regions. *Cx40* RNA was visualized by WM-ISH. The middle panel: *Cx40* expression in the left embryonic femoral tract at H&H stage 35, which represents the development of feather buds in the midline of the spinal tract between H&H stage 31+ and 34. Lower panels: after WM-ISH, the embryos were embedded in paraffin and then longitudinally sectioned along or near the midline of the spinal tract. Tissue sections were counter-stained with 10% eosin Y. Thickness of tissue sections: 20 μm. A, anterior. P, posterior. Scale bars, 300 μm (whole embryo); 50 μm (tissue sections). (B) Expression of the listed connexin isoforms shown by WM-ISH (left panels) and longitudinal sections along or near the midline of the chicken embryo spinal tract (right panels) at the stages between H&H stage 31 and 35. A, anterior. P, posterior. Scale bars, 300 μm (whole embryo); 50 μm (tissue sections). WM-ISH, whole-mount in situ hybridization.

*Connexin 40* exhibited dotted patterns in the epithelium at H&H stage 31 to 35 and was undetected at earlier stages (Fig 4A). At H&H stage 31, *Cx40* was evenly expressed and then disappeared in the bud domain. From H&H stage 34 to 35, *Cx40* expression was enhanced in the proximal posterior end and gradually covered the entire feather bud epithelium (Fig 4A). This transitional expression pattern could be best observed in the femoral tract (Fig 4A, middle panel). The dotted patterns suggest that *Cx40* was associated with specific cellular activities or cell types. Indeed, studies have shown that connexin 41.8, an ortholog of chicken Cx40 in the zebrafish, could regulate stripe/spot patterning [42,43]. In Japanese quails, melanocyte Cx40 expression could modulate body pigment stripe formation [44]. These studies strongly suggest that *Cx40* was expressed in chick embryo precursor pigment cells.

**Fig 5:**
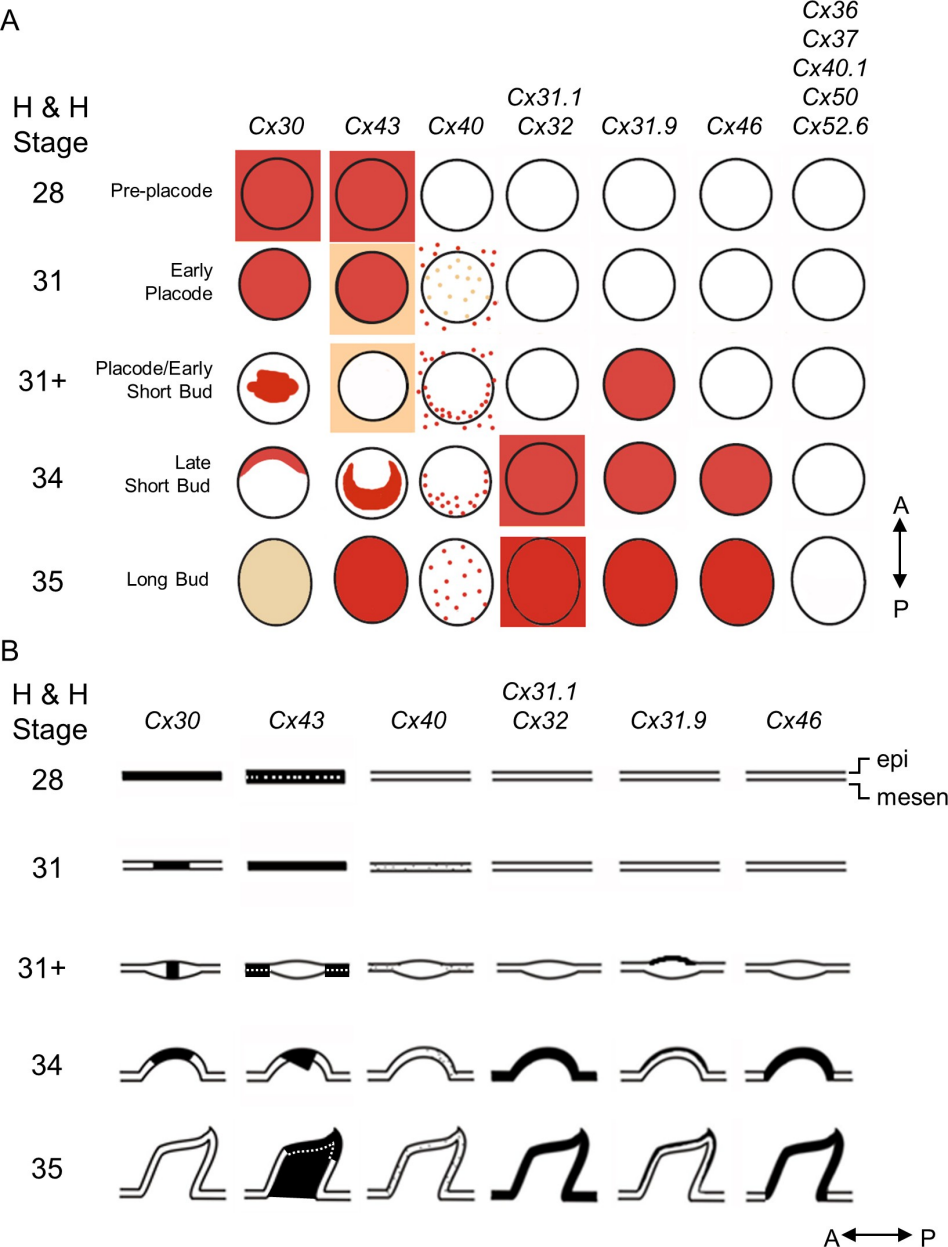
Schematic summary of connexin expression patterns during feather bud development. (A) Viewed from the top. *Cx30* was expressed early in the emerging feather tract at H&H stage 28 and later (H&H stage 31) became restricted to the bud primordia. The expression gradually disappeared in the marginal regions of buds (H&H stage 31+) and eventually appeared in anterior bud regions (H&H stage 34). Expression was nearly undetectable at long bud stage (H&H stage 35). *Cx43* was expressed in the feather-forming field (H&H stage 28) and gradually increased in the bud domain (H&H stage 31) but was completely gone at the early short bud stage (H&H stage 31+). At H&H stage 34, expression in the interbud domain disappeared while expression became intensified at more posterior side of the buds. Then, (H&H stage 35) the signal occupied the entire bud domain. The remaining Cxs did not appear at initial stages (H&H stage 28). Among them, *Cx40* shows a stippled pattern around the entire epithelium at St 31. This expression disappears transiently within the bud but gradually reappears in the posterior bud domain. At approximately the same time, the expression in interbuds was markedly lost. At H&H stage 35, *Cx40* was expressed in entire bud domain with only sporadic expression detectable in the interbud. *Cx31*.*1* and *Cx32* were expressed with similar patterns in the bud and interbud domains starting from H&H stage 34 and beyond. *Cx31*.*9* and *Cx46* were expressed in bud domains starting from H&H stage 31+ and 34, respectively. *Cx36*, *Cx37*, *Cx40*.*1*, *Cx50*, and *Cx52*.*6* were undetectable between H&H stage 28 and 35. A, anterior. P, posterior. (B) The schematic diagrams show the dynamic connexin expression in the sections after WM-ISH. A, anterior. P, posterior. epi, epithelium. mesen, mesenchyme. WM-ISH, whole-mount in situ hybridization.

Other connexin isoforms, including *Cx31*.*1*, *Cx32*, *Cx31*.*9*, and *Cx46*, were detected by WM-ISH at later stages (Fig 4B). The expressions of *Cx36*, *Cx37*, *Cx40*.*1*, *Cx50*, and *Cx52*.*6* were undetected. Both the *Cx31*.*1* and *Cx32* were weakly expressed, if expressed at all, at H&H stage 34 and strongly expressed in both bud and interbud epithelium at H&H stage 35. *Cx31*.*9* expression could be identified at H&H stage 31 and afterwards in the outer bud epithelium. In the bud epithelium, Cx46 was weakly expressed at H&H stage 34 and more strongly expressed at H&H stage 35. These differential expression patterns in different bud and interbud epithelium layers suggest the roles that these connexins might play in epidermal differentiation and compartmental development. The schematic illustration in Fig 5 summarizes the expression patterns of all the connexins we studied, viewed from above the embryos (Fig 5A) or observed in tissue sections (Fig 5B). Collectively, these dynamic connexin expression patterns during feather morphogenesis suggest that they are active participants in early feather patterning. The distinct and overlapping patterns indicate connexins may have unique and shared functions during feather morphogenesis.

### Connexin 30 is required for feather bud formation during early skin development

**Fig 6:**
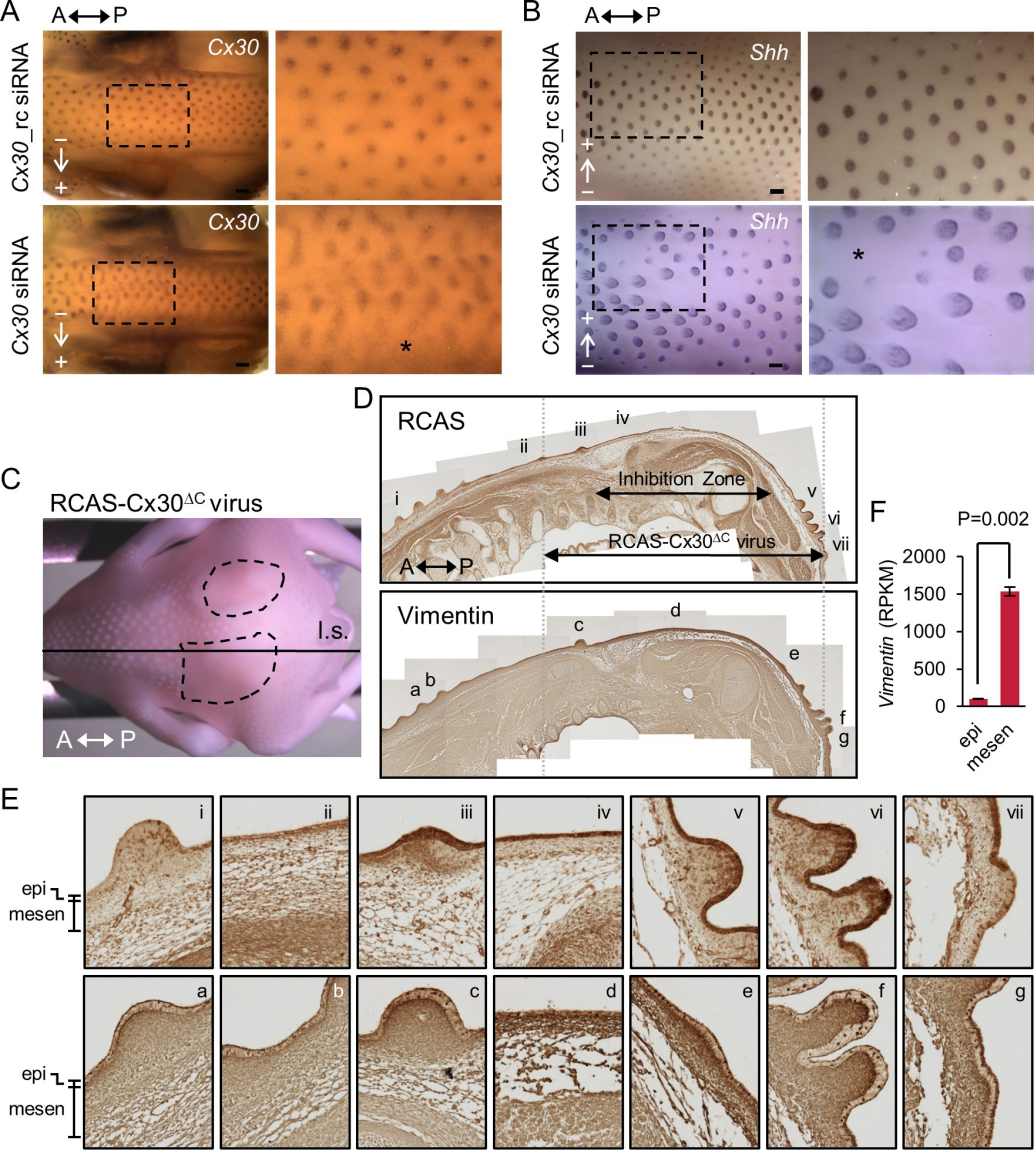
Functional perturbations of connexin 30 by siRNA and a DN mutant suppress the formation of feather buds. (A) *Cx30* siRNA and the randomized control (rc) were injected and then electroporated in ovo into embryos at H&H stage 26. The embryos were collected at H&H stage 31 and stained with probes against *Cx30* by WM-ISH. Arrows indicate the direction of siRNA flow. Boxed regions were enlarged on the right panels. The asterisk shows the inhibition zone. *n* = 2 for each condition. A, anterior. P, posterior. Scale bars, 300 μm. (B) *Cx30* siRNA and the randomized control (rc) were injected and then electroporated in ovo into embryos at H&H stage 26. The embryos were collected at H&H stage 35 and stained with probes against *Shh* by WM-ISH. Arrows indicate the direction of the siRNA flow. Boxed regions were enlarged on the right panels. The asterisk shows the inhibition zones. *n* = 4 for each condition. A, anterior. P, posterior. Scale bars, 300 μm. (C–E) RCAS viruses carrying Cx30 (a.a. 1–214) lacking the COOH-terminal cytosolic region (RCAS-Cx30^ΔC^) were in ovo injected into amniotic cavities of embryos at H&H stage 16/17 (*n* = 2). (C) The RCAS-Cx30^ΔC^ replication-competent virus-infected embryo was collected at H&H stage 35 and then paraffin-embedded and longitudinally sectioned (l.s.) along the indicated line. The dashed circles mark the inhibition zones. A, anterior. P, posterior. (D) IHC was performed using the antibodies against RCAS viruses (upper panel) and Vimentin (lower panel) followed by the 3, 3′-diaminobenzidine (DAB) substrate to develop the color (brown). The images of the entire tissue section were manually photo-stitched using Photoshop CS2 software. The double arrows indicate the inhibition zone showing no feather primordia growth and the area with detectable RCAS viruses in the epithelium, respectively. The approximate virus-infected regions are also marked by the dashed lines across both images. The areas near the marked regions (i–vii and a–g) were enlarged in panel E. A, anterior. P, posterior. (E) The enlarged images show the results of IHC from the indicated areas of panel D. Note that RCAS and Vimentin expressions are in darker brown. epi, epithelium. mesen, mesenchyme. (F) Reorganization of the previously deposited RNA-seq results (GSE86251) of the gene expression of *Vimentin* in the epithelium (epi) or mesenchyme (mesen) of embryos at H&H stage 31. *p*-value (*P*) = 0.002 by a two-tailed and unpaired Student’s *t* test. DN, dominant-negative; IHC, immunohistochemistry; RCAS, replication-competent avian sarcoma-leukosis virus long terminal repeat with splice acceptor; WM-ISH, whole-mount in situ hybridization.

We observed that *Cx30* expression is highly dynamic, and it is among the earliest detected GJ isoforms during early feather patterning. Additionally, its functions are still largely unknown compared to another abundantly expressed GJ isoform, *Cx43*. Therefore, we further investigated its functional importance by siRNA and the avian sarcoma-leukosis retrovirus RCAS (replication-competent avian sarcoma-leukosis virus long terminal repeat with splice acceptor)-mediated overexpression of Cx30 deletion mutant (a.a. 1–214) lacking the COOH-terminal region (Cx30^ΔC^) (Fig 6). We found that perturbation of Cx30 expression by siRNA through in ovo electroporation in embryos at H&H stage 26 resulted in suppression of feather bud formation in chick embryos collected at both St31 (Fig 6A, asterisk) and St35 (Fig 6B, asterisk). A randomized siRNA sequence was used as the control. *Shh* is the feather bud marker expressed in the distal bud tip and the marginal plate epithelia of feather filaments. These results suggest that Cx30 is required for the feather bud formation during early feather patterning.

We next introduced replication-competent RCAS-Cx30^ΔC^ viruses into the chick embryo at H&H stage 16/17 by in ovo injection into the amniotic cavity (Fig 6C). The RCAS-Cx30^ΔC^ virus-infected embryo exhibited inhibition of feather bud formation at H&H stage 35 (Fig 6C, the areas marked by dashed circles). The presence of RCAS viruses was confirmed by immunohistochemistry (IHC) using the antibody against viral capsid protein p27 in the longitudinally sectioned (l.s.) paraffin-embedded embryo (Fig 6D and 6E, upper panels). We found that the RCAS viruses infected mostly the epithelium (Fig 6E, upper panels; in darker brown), and the infected area (as shown in Fig 6E, iii to vii) was larger than the zone of inhibition in the tissue section (Fig 6D, upper panel). These results indicate that the RCAS-Cx30^ΔC^ viruses can act dominant-negatively to suppress feather formation, and this process is more effective when a higher concentration of viruses and/or an earlier viral infection occurs in the area (as the RCAS viruses are replication-competent, they can spread out from the initially infected areas during development). These observations, together with the results of siRNA experiments, confirm the importance of Cx30 in early feather bud development. We next tried to explore the molecular mechanism of the observed inhibition. Therefore, we performed IHC using the antibodies targeting a concise list of candidate proteins in adjacent tissue sections from the RCAS-Cx30^ΔC^ virus-infected embryo and found the protein level of Vimentin was elevated in the epithelium of the inhibition zone (Fig 6E, panel d in the lower panels) compared to the nearby regions infected by the viruses (Fig 6D and 6E, lower panels). The previous study [38] showed that the RNA or protein level of Vimentin was predominantly expressed in dermal fibroblasts in developing feather buds at H&H stage 31 to 35. We reorganized their RNA-seq results available from the GEO dataset, GSE86251, for the embryos at H&H stage 31 and presented them in Fig 6F. The results that we obtained from the RCAS-Cx30^ΔC^ and Vimentin experiments suggest that Cx30 may facilitate the specification of epithelium during early feather patterning, and this warrants further thorough investigation.

### Functional coupling of gap junctions during early feather patterning

The expression of connexin isoforms does not necessarily indicate that they can form functional gap junctions allowing direct communications between neighboring cells. Previously, our lab visualized GJIC in embryonic chicken skins by injecting LY into a single cell and observed efficient LY dye transfer between cells in the H&H stage 30 to 32 embryonic placode epithelium [41]. In the same study, our lab also observed efficient LY dye coupling in the mesoderm but less so in the epithelium of short buds (equivalent to H&H stage 35 in this study). Dye transfer in the interbud epithelium at this stage is moderately efficient. Notably, LY dye transfer does not cross the bud epithelium and mesoderm boundary, nor the boundary of bud and interbud regions at the short bud stage.

**Fig 7:**
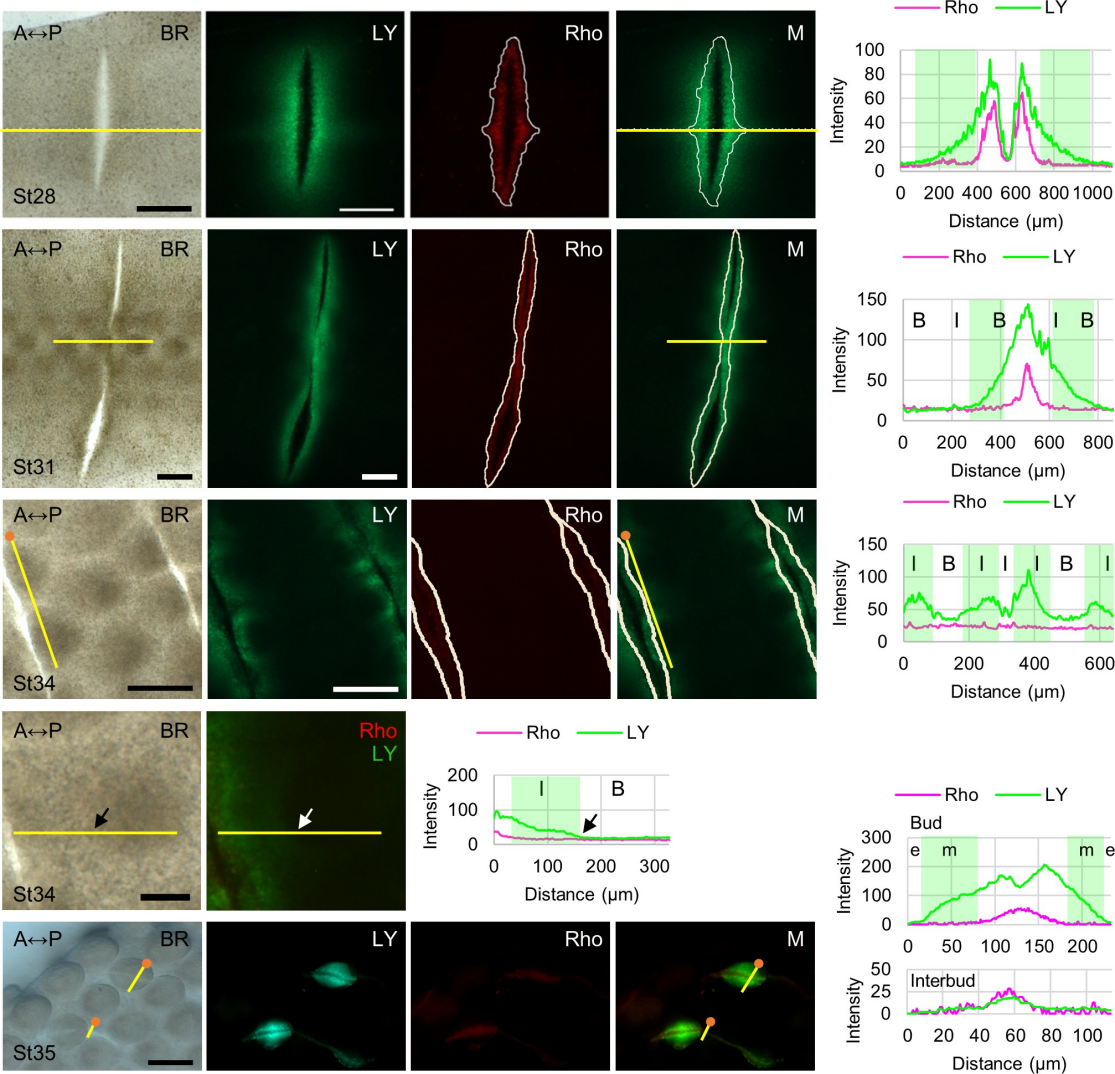
GJIC visualized by scrape-loaded LY dye transfer assay in H&H stage 28–35 chicken dorsal skins. Left panels: bright-field (BR) and epifluorescent micrographs showing LY dye transfer (green) in the St28 (*n* = 2), St31 (*n* = 3), St34 (*n* = 4), or St35 (*n* = 3) skin explants. Rhodamine dextran (Rho; 10 kDa, in red) is too big to pass through GJs and served as a control for the wound caused by the scrape-loading procedure. The solid white lines demarcate the edge of the rhodamine dextran signal. The merged (M) images were created by overlaying the Rho signal or the edge of the Rho signal (the white line) onto the LY signal. The rightmost panels show the X-Y scatter plots of the fluorescence intensities of LY and Rho that were measured along the yellow lines from the skin explants shown on the left by Image J software, and the results were processed by Excel. The approximate ranges of LY dye transfer were marked in a lighter green color in the X-Y scatter plots. Distance represents the length of the yellow lines, starting from the anterior side or the orange dot. The triangle-headed arrows at St34 indicate the same position. A, anterior. P, posterior. B, bud domain. I, interbud domain. Scale bars, 300 μm. Raw data of the measurements is available in S1 Data. GJIC, gap junctional intercellular communication; LY, lucifer yellow.

Here, we expanded this study and utilized previously described [45] scrape-loading of LY to visualize macro-scale GJIC in the spinal tract during early feather morphogenesis in the skin explants (Fig 7 and S1 Data, H&H stage 28 to 35). We did not differentiate the localizations of LY dye transfer occurring in the epithelium or the mesoderm when we performed epifluorescence imaging due to the limitations of our imaging settings. We found that at pre-placode H&H stage 28, LY dye transfer (in green) efficiently crosses the dorsal skin compared to the dye loading sites indicated by the rhodamine dextran (Rho, in red; 10 kDa) signal. The intensities of LY and Rho along the yellow line were measured in Image J software and used to create an X-Y scatter plot in the rightmost panel (Fig 7, the first row). At H&H stage 31, the initial few rows of feather primordia start to form, and the boundary between the bud and interbud becomes visible in the first row of the feather primordia (Fig 7, the second row). We found that dye transfer occurs in both the bud domain and the interbud domain in the first row. At H&H stage 34 (Fig 7, the third and fourth rows), short feather buds exhibit apparent A-P polarity and start to elongate. We observed that LY dye transfer is more effective in the interbud region and is gradually reduced towards the center of the bud when the dye was directly loaded into the bud or the interbud region (Fig 7, the third row). We also found that dye transfer was reduced near the center of the interbud region. These results suggest that there could be a physical or molecular barrier between the buds. To support this notion, we showed that the dye loaded into the interbud region could not be transferred into the bud (Fig 7, the fourth row; arrows indicate the visible boundary of the bud and the interbud region). At H&H stage 35, the elongating feather bud exhibited efficient LY dye coupling in the bud mesoderm (Fig 7, the fifth row). Dye transfer in the bud epithelium was extremely limited and was not evident in the interbud region. These results suggest that there are physical or molecular barriers between bud epithelium and mesoderm as well as the bud and the interbud region. Taken together, these results show that GJIC is tightly regulated in both a temporal and spatial manner, and it reflects a dynamic landscape/compartmentalization during early feather morphogenesis, suggesting that GJs facilitate the establishment of boundaries between different compartments. Further investigation is required to unravel GJ isoform-specific regulations in GJIC during feather patterning.

### Suppression of GJIC activities allows the sequential emergence of ectopic feather primordia

**Fig 8:**
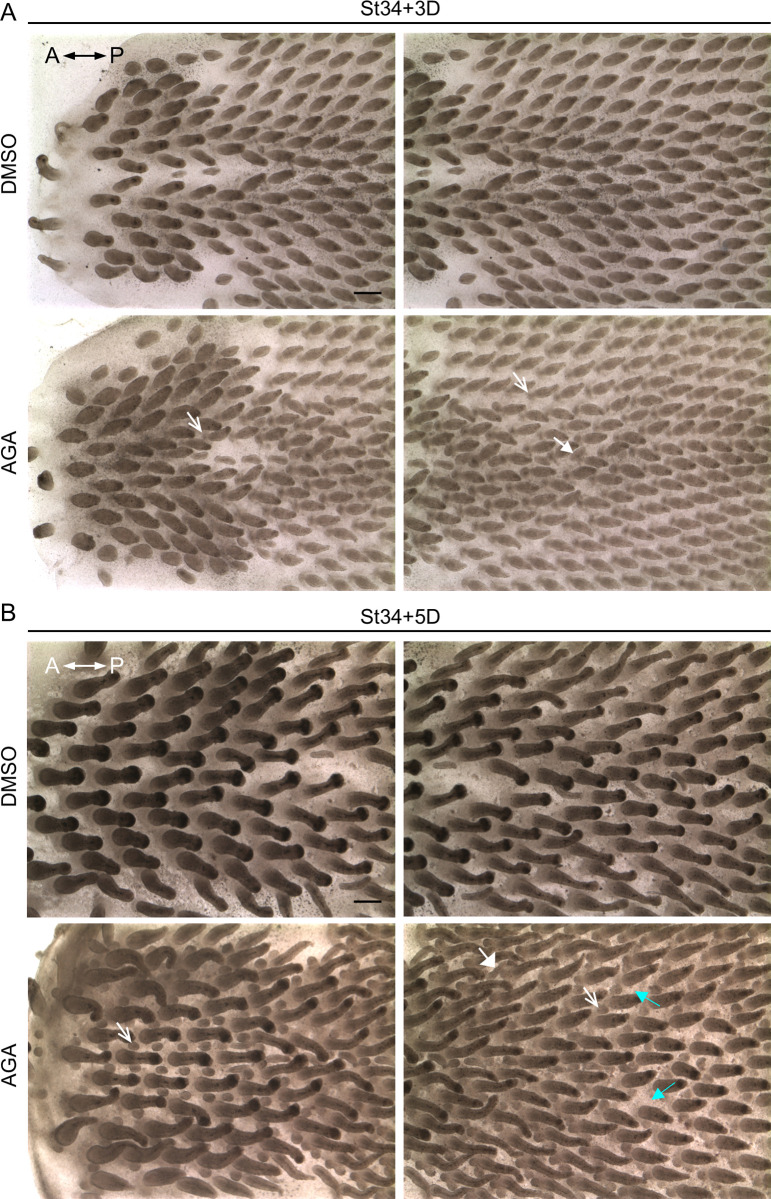
Inhibition of GJIC by 18 AGA stimulates the formation of ectopic feather buds. (A, B) Bright-field micrographs showing H&H stage 34 skin explants treated with AGA or DMSO control for 3 (A) or 5 (B) days. The arrows indicate the ectopic feather buds localized around the base of the primary feather buds. The triangle-headed arrows (in white) show the ectopic buds localized in the interbud regions. The triangle-headed arrows (in cyan) point to the space around the primary buds. A, anterior. P, posterior. Scale bars, 300 μm. AGA, α-glycyrrhetinic acid; GJIC, gap junctional intercellular communication.

**Fig 9:**
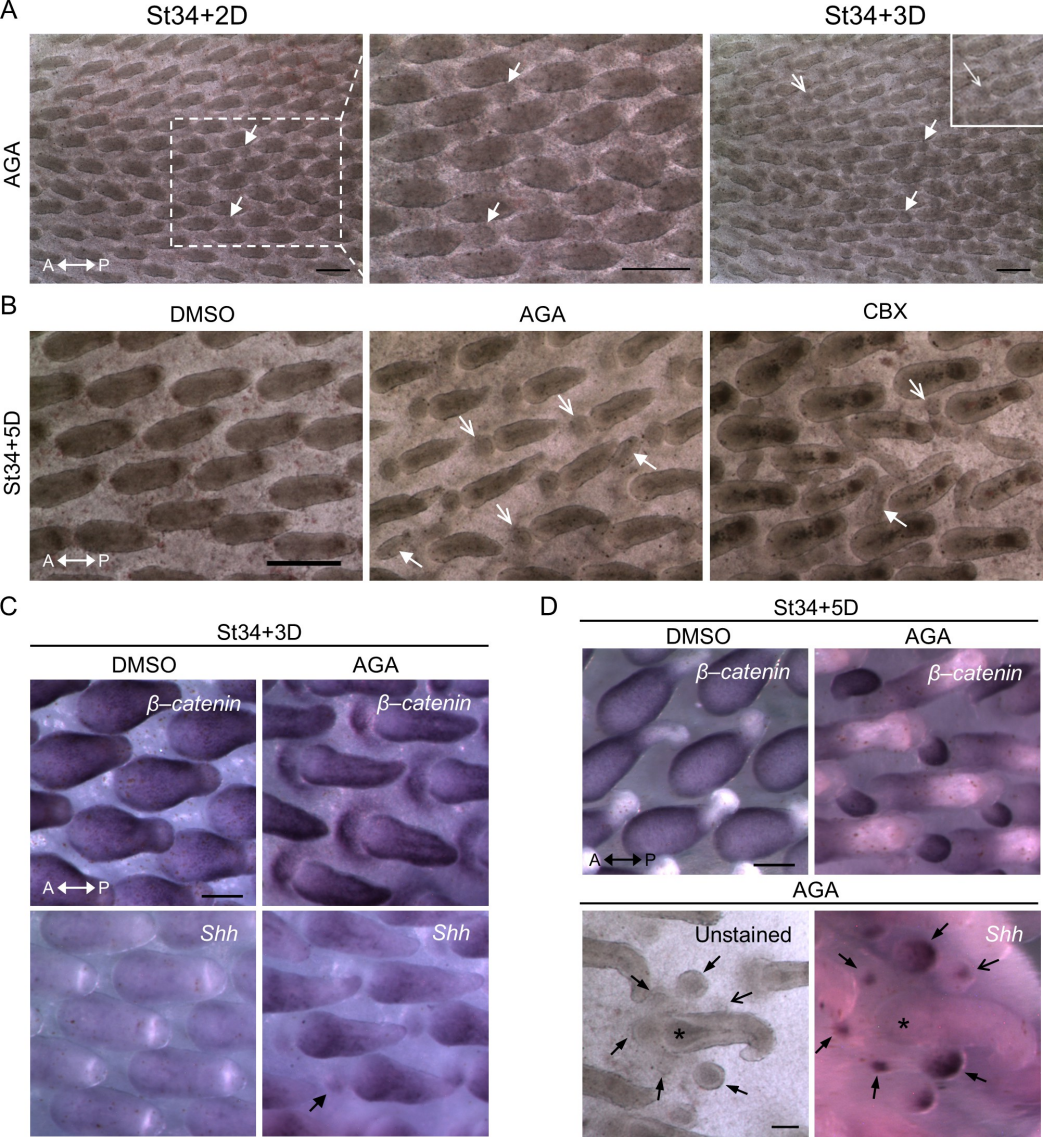
Ectopic feather buds showed molecular markers of feather primordia. (A) Bright-field micrographs showing the H&H stage 34 skin explant treated with AGA for 2 and 3 days. The boxed region in the leftmost panel was enlarged in the middle panel. The triangle-headed arrows indicate the ectopic feather buds localized at comparable sites in the interbud regions. The inset within the rightmost panel shows an enlarged image of the area around the base of the primary feather bud that are pointed out by the arrows. A, anterior. P, posterior. Scale bars, 300 μm. (B) Bright-field micrographs showing H&H stage 34 skin explants treated with DMSO control, AGA or its derivative, carbenoxolone (CBX), for 5 days. The arrows show the ectopic feather buds localized around the base of the primary feather buds. The triangle-headed arrows indicate the ectopic buds localized in the interbud regions. A, anterior. P, posterior. Scale bar, 300 μm. (C, D) WM-ISH of embryonic chicken dorsal skin explants treated with AGA or DMSO control. H&H stage 34 skins were harvested and then ex vivo cultured for 3 (C) or 5 (D) days. The probes for in situ hybridization targeted *β-catenin* and *Shh*, the early transcriptional markers of feather primordia formation. A, anterior. P, posterior. Scale bars, 100 μm. (C) The triangle-headed arrow indicates the expression of *Shh* at the distal tip of the feather bud. (D) The arrows and triangle-headed arrows indicate ectopic feather buds localized at comparable sites around the primary feather bud (asterisk). AGA, α-glycyrrhetinic acid; WM-ISH, whole-mount in situ hybridization.

We next investigated the functional importance of GJIC during early feather morphogenesis by inhibiting GJIC in H&H stage 34 chicken dorsal skin explants with AGA [46–48]. The continuous presence of AGA induced ectopic buds in culture compared to the DMSO control (Fig 8). The bright-field images show the upper thoracic regions (left panels) and the lower thoracic regions (right panels). By day 3 in culture, ectopic buds appeared at the anterior around the base of primary feather buds (arrows) and interbud regions (triangle-headed arrow) (Fig 8A). The size of the ectopic buds became bigger on day 5 (arrows and triangle-headed arrow, in white), and a variety of ectopic bud localizations appeared around the base of primary feather buds (Fig 8B). Interestingly, we rarely observed ectopic buds localized towards the midline around the base of primary feather buds flanking the lower thoracic region. The absence of ectopic buds was highlighted by the triangle-headed arrows (in cyan). The upper and lower thoracic regions also displayed differential preferences of ectopic bud localizations. The ectopic buds in the upper part tended to appear at both right and left sides around the base of primary feather buds, and the lower part favored the anterior and/or the right or the left side (arrows). The observed regional differences suggest that additional factors may influence the capacity of local tissues to respond to the AGA treatment. Some potential candidate factors are cell density and tissue mechanics [9,49]. Additionally, we observed that the ectopic buds localized in the interbud regions (Fig 9A, triangle-headed arrows) appeared at day 2 in culture, about 24 h earlier than the ectopic buds localized at the anterior around the base of primary feather buds (Fig 9A, arrows). To validate the effectiveness of AGA in the inhibition of GJIC, we performed a scrape-loaded LY dye transfer assay and observed greatly reduced LY dye transfer upon AGA treatment compared to the DMSO control (A in S2 Fig). GJIC was quantified by measuring the intensity of LY transfer away from the edge of damaged cells (A in S2 Fig, right panel, and S1 Data). A nonfunctional synthetic analog of AGA, glycyrrhizic acid, was used to verify the specificity of AGA, and it does not induce ectopic feather buds (B in S2 Fig). Additionally, we found that carbenoxolone (CBX) [47,48,50], the hemisuccinate derivative of AGA, treatment induces ectopic buds similar to the AGA treatment (Fig 9B, arrows and triangle-headed arrows). The ectopic buds express correct patterns of transcriptional markers of feather buds as demonstrated by the whole-mount in situ hybridization using the RNA probes targeting *β-catenin* (*β-catenin* expression in the feather tract and developing bud) or *sonic hedgehog* (*Shh*; *Shh* expression at the tip of the bud and the marginal plate of developing feather filament) (Fig 9C and 9D) [8,51–53]. Notably, by day 3 in culture, *β-catenin* expression was detected as a crescent shape at the anterior, extending to the bilateral regions around the primary feather buds (Fig 9C). We observed a condensed expression of *β-catenin* at the anterior bud, which is similar to where *Shh* was expressed. This indicates that the anterior has started to form a feather primordium. The expression of *β-catenin* was then restricted to the bud area as the ectopic buds further developed (Figs 9D and S2C). Interestingly, we found that evenly spaced ectopic buds, as demonstrated by the *Shh* staining, appeared along the crescent path similar to where *β-catenin* was expressed (Fig 9D, lower panels; arrows and triangle-headed arrows show the ectopic buds at comparable sites before and after staining). These results point to an intriguing possibility that GJIC inhibition modifies local tissue competence and allows the formation of new patterns, and GJ may mediate the exchange of inhibitory factor(s) between neighboring cells to suppress the emergence of ectopic feather buds during normal development.

The earliest detected GJ isoforms, *Cx30* and *Cx43*, are expressed before the formation of feather primordia (Figs 2 and 3) when GJIC can already be detected by the LY dye transfer assay (Fig 7, the first row). Therefore, we next investigated the consequences of GJIC inhibition at the earlier pre-placode stage (H&H stage 28) during feather patterning (S3 Fig). Similar to the later stage (Fig 8), we found that the primary feather array still formed with the expected hexagonal patterns (highlighted in dash lines), and the AGA treatment also stimulated the formation of ectopic buds (arrows) compared to the DMSO control.

### Summary and quantitative description of bud localizations upon GJIC inhibition

**Fig 10:**
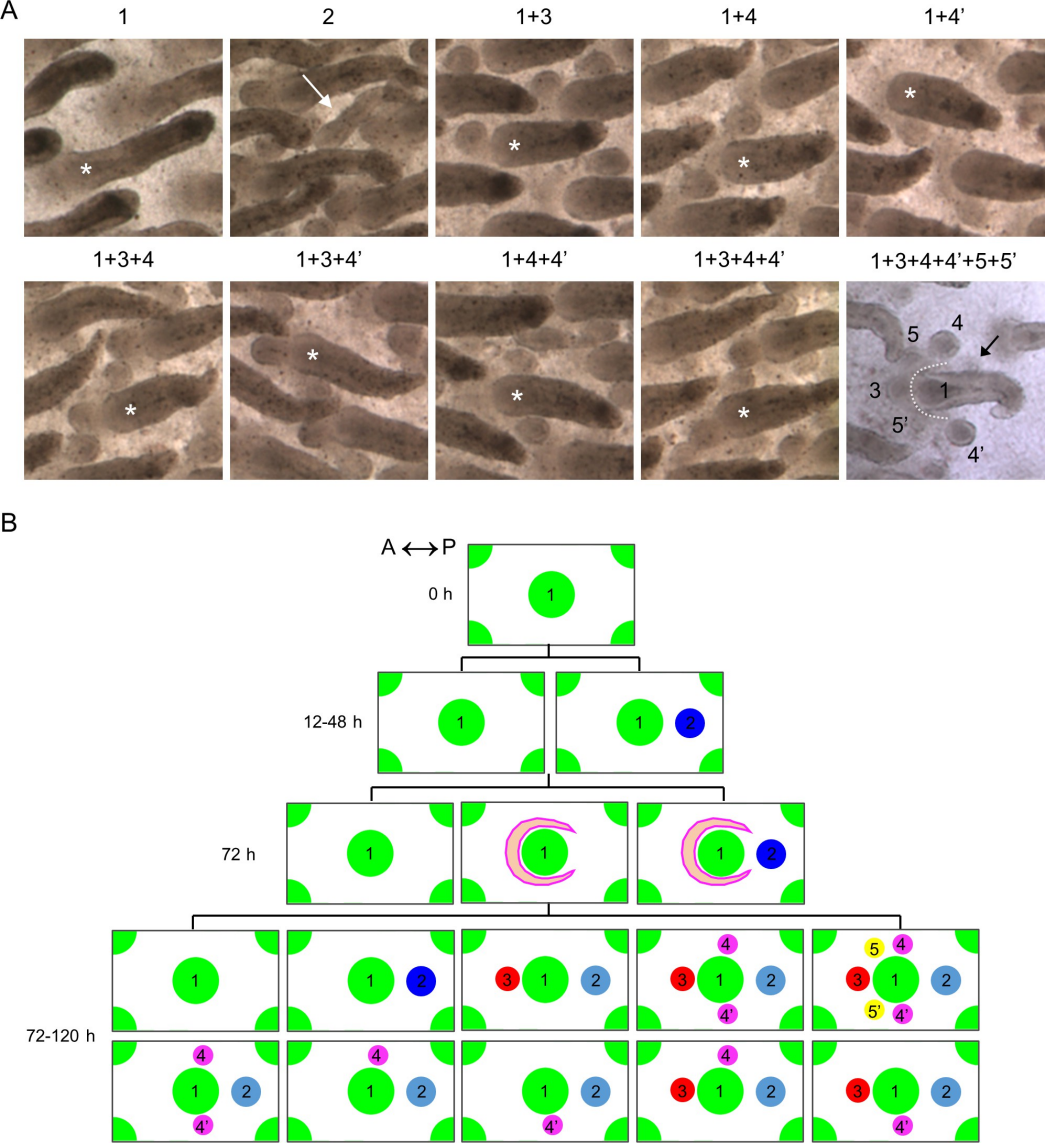
Summary of observed sites of primary and ectopic feather primordia in H&H stage 34 skin explants treated with the GJIC inhibitor. (A) Bright-field micrographs showing examples of bud combinations from the H&H stage 34 skin explant treated with AGA for 5 days. The numbers represent the spatial location of the feather primordia with spot numbered one or marked with an asterisk as the primary bud. Spots numbered 2 to 5 represent the ectopic buds. The triangle-headed arrow (in white) indicates the ectopic bud localized at the interbud region. The triangle-headed arrow (in black) shows an ectopic bud localized at a rarely observed location around the primary bud. The dotted line highlights the area showing ectopic buds around the anterior of the primary bud. (B) Schematic showing of the spatiotemporal emergence of the primary and ectopic feather buds. The ranked numbers represent the temporal order and the spatial location of the feather primordia with spot numbered one as the earliest and the primary bud. Spots numbered 2 to 5 represent the ectopic buds. Time 0 h, starting the ex vivo culture of H&H stage 34 skin explants. Crescent shapes indicate the emerging field competent to form new buds. Spots in dark or light blue are localized at the same spatial position in the interbud region. The lighter color represents a more flexible state to appear or not to appear in the specific combination of buds as shown in the image. h, hour. A, anterior. P, posterior. AGA, α-glycyrrhetinic acid; GJIC, gap junctional intercellular communication.

Our experiments showed that the AGA treatment stimulated spatiotemporal emergence of ectopic feather buds (Figs 8 and 9). Interestingly, we also found that the primary and ectopic buds could appear in multiple combinations of spatial locations, and we summarized our observations in Fig 10A with the numbers indicating specific spatial locations as shown on the images. The primary bud is numbered one or marked with an asterisk if it is included in the indicated combination. The triangle-headed arrow (in white) indicates the ectopic bud localized at the interbud region (spot 2). The ectopic bud localized at spot 2 grows longer than the ectopic buds localized at other spatial locations because it appears earlier. The lower right panel shows an example of ectopic buds that emerged at all observed spatial locations around the base of the primary bud. The triangle-headed arrow (in black) points out an ectopic bud localized at a rarely observed location around the primary bud. The dotted line highlights the area formed by the ectopic buds (spots 3 to 5) similar to the crescent-shaped area showing elevated β-catenin expression at the earlier time point (Fig 9C). Notably, we did not observe any ectopic buds immediately posterior to the primary buds.

In the schematic summary (Fig 10B), we used the ranked order to indicate the temporal sequence of primary and ectopic bud appearance and their spatial locations as shown above. The first patterning cues create the primary buds is at spot 1. The ectopic buds residing at spot 2 are in the interbud region and show up early between days 1 and 2 in culture. The additional ectopic buds at other numbered locations start to grow in a crescent-shaped region at day 3 in culture and appear over a period of 24 to 48 h at the anterior and flanked regions around the base of primary buds. The ectopic buds at spot 3 are more frequently observed and show earlier expressions of the feather bud markers (Fig 9C) compared to the spots 4/4’ and 5/5’. The ectopic buds at spot 5/5’ are the least frequently found. The ectopic buds at spot 2 can coexist with buds at other locations but do not always appear.

To further quantify our observations of the spatial distributions of ectopic buds, we divided the thoracic-lumbar region of the dorsal skin into different anatomical subareas (S4A Fig, boxed regions). We chose these subareas for quantification because these regions provide clearer views of ectopic buds and enough feather buds across different biological samples. The box-and-whiskers plot shows the percentage of each bud combination observed in the indicated subareas (S4B Fig). We further performed Tukey’s multiple comparisons test and highlighted some of the test results showing differences in the regional-specific appearance of bud combinations (S4C Fig). From the results of bud quantifications and Tukey’s multiple comparisons test, we have made the following observations: (i) Ectopic buds are less likely to emerge in the middle area than the flanked regions (right and left areas); (ii) there is a higher probability to observe ectopic buds localized at spots 3 and 4 on the right side of the tissue, although this is not statistically significant; and (iii) there is a higher probability to observe ectopic buds localized at spots 3 and 4’ on the left side of the tissue. Next, we tried to visualize inter-sample heterogeneity in the emergence of ectopic buds localized at individual spatial locations regardless of the bud combinations (S4D Fig). We found that ectopic buds localized at spot 3 are more frequently observed in most of the subareas across the samples followed by spot 2 or 4/4’ and then spot 5/5’. Taken together, the observed regional differences further support our observations in Fig 8. The incidence of ectopic buds localized at individual spatial locations provides further clues about the temporal sequence of ectopic buds that supports our schematic summary (Fig 10B).

### Exploring the formative process of ectopic feather buds

**Fig 11:**
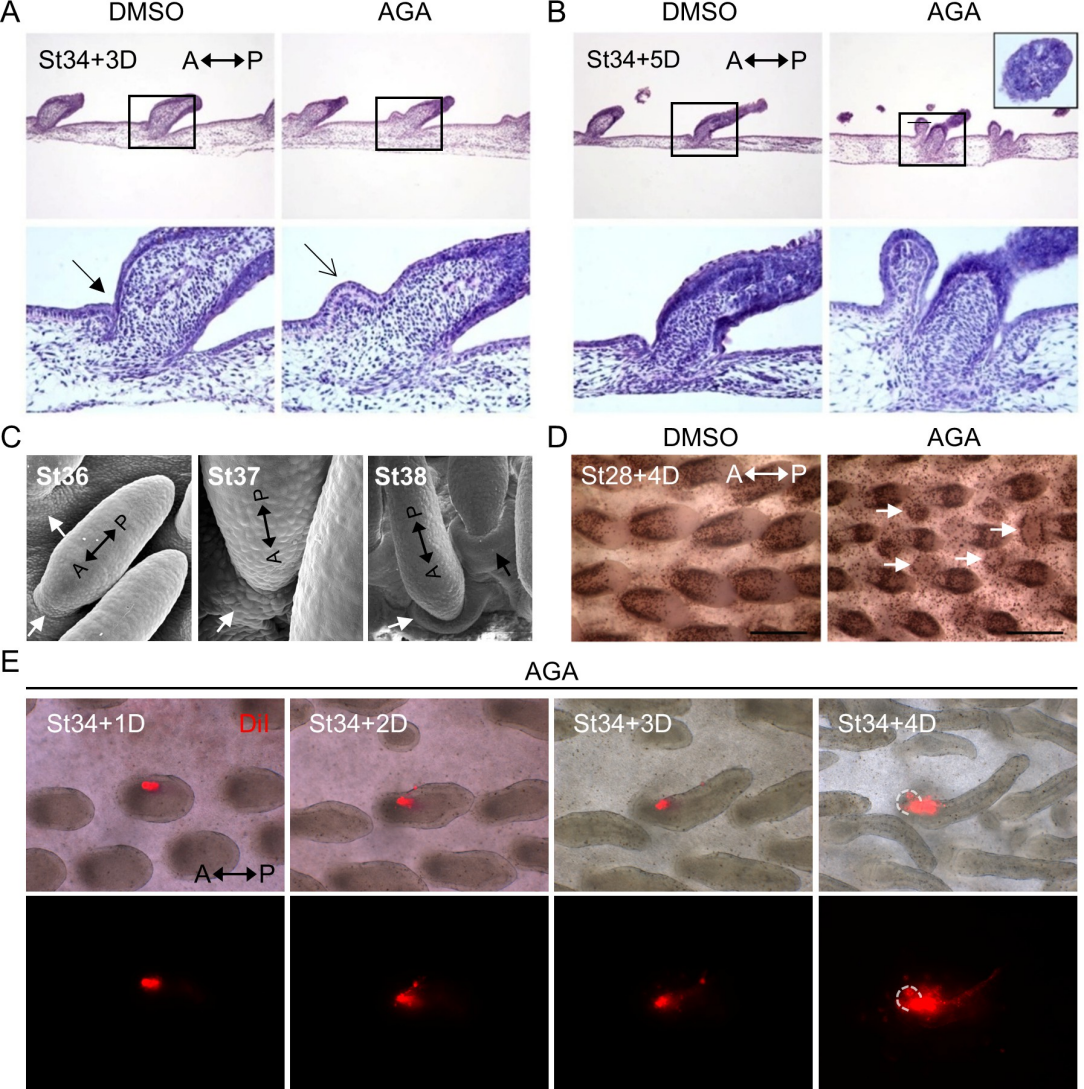
Ectopic feather buds exhibited structural characters of normal feather buds and enhanced cell proliferation. (A, B) HE staining of paraffin-embedded tissue sections obtained from H&H stage 34 skin explants treated with AGA or DMSO control for 3 or 5 days. Box regions near the center of the upper images from each day were enlarged and shown in the lower images. The triangle-headed arrow and the arrow indicate similar anatomical locations. The inset within the upper right panel shows an expanded growth along the cross-sectional plane after AGA treatment. A, anterior. P, posterior. (C) Scanning electron microscopic images showing in vivo elongated primary feather buds in the dorsal skins of chick embryos at H&H stages 36, 37, and 38. The triangle-headed arrows indicate the folding/invagination structures at the anterior of feather buds. A, anterior. P, posterior. (D) Bright-field micrographs showing the H&H stage 28 dorsal skin explants treated with AGA or the DMSO control for 4 days and then pulse-labeled with BrdU for 4 h followed by immunostaining with the anti-BrdU antibody. The triangle-headed arrows indicate ectopic feather buds. A, anterior. P, posterior. Scale bars, 300 μm. (E) The H&H stage 34 skin explants were harvested at day zero and treated with AGA, and then the primary feather buds were labeled with Vybrant DiI at 25 μm (Invitrogen, in red) at day 1 of the ex vivo culture. Snap shots of the bright-field and fluorescent images were taken each day after labeling for 4 days. Dashed lines indicate the ectopic feather bud. Upper panels, merged bright-field, and DiI images. Images were processed in the ImageJ software. Lower panels, DiI signals. Four out of 7 successfully labeled primary feather buds from 2 biological replicates show the Dil signal in the ectopic buds. A, anterior. P, posterior. AGA, α-glycyrrhetinic acid; HE, hematoxylin and eosin.

To further confirm the identity of ectopic feather buds, we performed paraffin-embedded tissue sectioning and hematoxylin and eosin (HE) staining on the H&H stage 34 skin explants treated with AGA or the DMSO control for 3 or 5 days (Fig 11A and 11B). We focused on the area localized at the anterior side around the base of the primary feather buds (spot 3) because we can unequivocally identify its location on tissue sections. The chicken embryonic epithelium starts to invaginate into the mesoderm at around H&H stage 37 [54], equivalent to H&H stage 34 plus 3 days in culture, and then forms feather follicles. We found that invagination of the epithelium (Fig 11A, triangle-headed arrow) did not occur in the AGA-treated skin explants compared to the DMSO control, and this led the epithelium to bulge out (Fig 11A, arrow). This structure keeps growing and starts to show early signs of branching morphogenesis at day 5 in culture, as shown in the enlarged cross-sectional image (Fig 11B). These histologies show that the epithelial bulged structure exhibits traits of true feather buds and can further differentiate into advanced feather structures. A closer look at the in vivo tissue structure around the H&H stage 36 to 38 chicken embryo dorsal feathers by scanning electron microscopy confirmed a similar folding/invagination structure observed in the H&H stage 34 skin explants after an additional 3 days of culture (Fig 11C, triangle-headed arrows). These results indicate that the observed developmental processes in the ex vivo cultured skin explants have functional relevance to the in vivo condition.

Next, we sought to determine whether cell proliferation and the primary bud contribute to the formation of ectopic feather buds. We utilized short-term (4 h) bromodeoxyuridine (BrdU) labeling and whole-mount immunostaining using the antibody against BrdU to visualize proliferating cells in the AGA or DMSO-treated skin explants (Fig 11D). For this experiment, we utilized H&H stage 28 (pre-placode stage) skin explants cultured for 4 days due to the improved antibody accessibility for whole-mount immunostaining compared to the H&H stage 34 skin explants. We found that the ectopic feather buds exhibit increased cell proliferation upon AGA treatment (Fig 11D, triangle-headed arrows). We then used DiI (in red) labeling to follow cell lineage and found that the cells initially localized in the primary feather bud can contribute to the cell population in the ectopic buds (Fig 11E). These results indicate that the emergence of ectopic feather buds can be attributed to localized cell proliferation and, at least in part, re-localization of cells from the primary feather buds.

**Fig 12:**
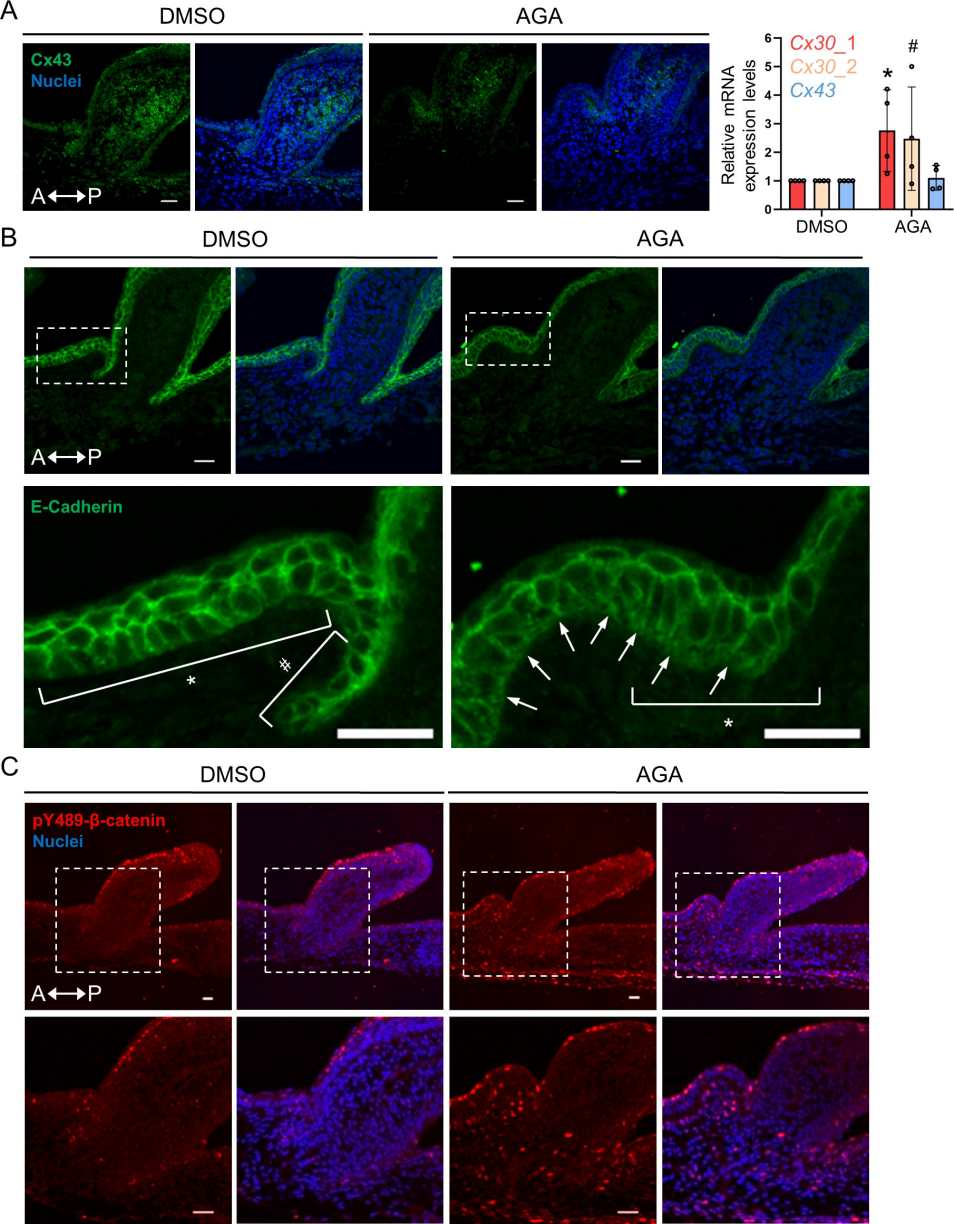
Characterization of ectopic feather buds. (A–C) Immunofluorescence and confocal micrographs showing the expression of the indicated proteins from paraffin-embedded tissue sections of H&H stage 34 skin explants treated with AGA or DMSO (control) for 3 days. A, anterior. P, posterior. (A) Left panels: The Cx43 antibody was used for immunostaining (green). Nuclei were stained by DAPI (blue). Scale bars, 20 μm. Rightmost panel: RT-qPCR analysis shows the mRNA expression of *Cx30* and *Cx43* in the skin explants harvested from the chick embryo at H&H stage 31 and then treated with AGA or DMSO control for 36 h. Data represents mean ± SD from 4 biological replicates. Each biological replicate has 2 qPCR experimental repeats. Statistical analysis was performed with a two-sample Student’s *t* test. *, *P* ≤ 0.05. #, *P* = 0.061. Raw data of the qPCR results is available in S1 Data. (B) The antibody against E-Cadherin was used for the immunostaining (green). Nuclei were stained by DAPI (blue). Boxed regions were enlarged below. Asterisks, columnar cells. Pound sign, cuboidal cells. Triangle-headed arrows, the basement membrane. Scale bars, 20 μm. (C) The antibody against phosphorylated β–catenin at tyrosine residue 489 (pY489–β–catenin) was used for immunostaining. Boxed regions were enlarged below. Phospho–β–catenin (red). Nuclei were stained by DAPI (blue). Scale bars, 20 μm for the upper panels; 50 μm for the lower panels. AGA, 18 α-glycyrrhetinic acid.

A closer molecular characterization of H&H stage 34 skin explants treated with AGA for 3 days revealed a substantial reduction of Cx43 protein expression compared to the DMSO control (Fig 12A, left panels). Interestingly, the AGA treatment did not lead to a change of *Cx43* RNA expression in 36 h compared to the DMSO control, instead, there was a 2- to 3-fold increase of *Cx30* expression (Fig 12A, rightmost panel). These results point out a possible role of Cx30 in the regulation of ectopic bud formation upon GJIC inhibition. Note that the RT-qPCR experiments were performed at an earlier time point than the immunostaining because we suspect that change in RNA levels would occur earlier than change in protein levels. Further studies are required to investigate the connections between Cx30 and Cx43 expressions and the underlying mechanisms of Cxs in ectopic bud formation. We then examined the epithelial tissue structure in H&H stage 34 skin explants treated with AGA or DMSO control for 3 days by confocal imaging of E-cadherin antibody immunofluorescence (Fig 12B). We found that the basal epithelial cells showed a “rough” membrane structure near the basement membrane upon AGA treatment (Fig 12B, triangle-headed arrows), suggesting an erratic composition of extracellular matrix and communication between basal epithelial cells and dermal cells. Additionally, we observed that there is a transition of basal cell shape from columnar cells (asterisk) to cuboidal cells (hash symbol) when the epithelium starts to invaginate into the mesoderm as shown in the enlarged image from the DMSO control (Fig 12B). In the AGA-treated skin explants, the basal epithelial cells remain columnar in shape (Fig 12B, asterisk in the bottom right panel), suggesting that the AGA treatment may perturb cell movement or modify the mechanical properties of the tissues. Furthermore, we discovered that the expression of transcriptionally active nuclear β-catenin, pY489- β-catenin [55], is elevated and accumulates in the ectopic feather bud (Fig 12C). As β-catenin was shown to stimulate ectopic feather formation [8], this result (pY489- β-catenin), together with the observed transcriptional activation of β-catenin from Fig 9C, suggests that AGA treatment re-initiates a developmental program of feather morphogenesis at both posttranslational and transcriptional levels. Moreover, the activation of β-catenin provides an additional layer of evidence of changes in local tissue mechanics as suggested by the previous study [13].

### Mathematical modeling of emerging new buds during feather pattern formation

**Fig 13:**
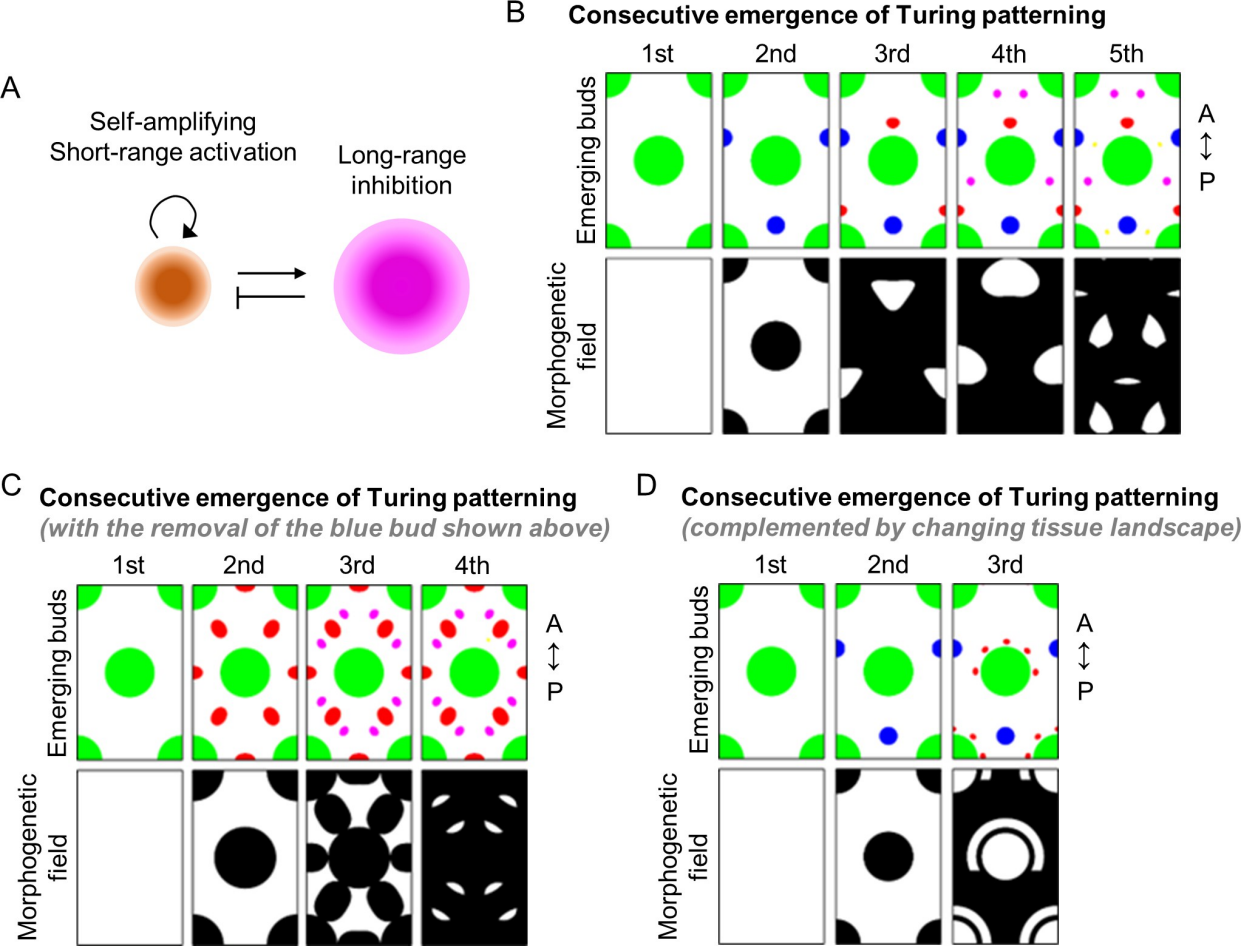
Mathematical modeling of the spatiotemporal emergence of ectopic feather buds. (A) Turing’s RD model. A slowly diffusing short-range activator can positively regulate its own production and that of a rapidly diffusing inhibitor. The latter in turn suppresses the production of the activator. (B) Top: Spatiotemporal emergence of ectopic buds. Bottom: Region with competence to undergo patterning is shown in white. Note the dynamic changes of competent regions due to the appearance of buds in the previous stage. All morphogens can diffuse through the domain; however, reactions can only take place in the white region. A, anterior. P, posterior. Animation is provided in S1 Movie. (C) The simulation is the same as that in (B), except spot 2 (blue bud in B) is removed from the simulation sequence. A, anterior. P, posterior. Animation is provided in S2 Movie. (D) Top and bottom represent buds and competent regions as described in (B). Here, we specify the horseshoe region around the first bud in which buds 3, 4/4’ and 5/5’ can appear. A, anterior. P, posterior. Animation is provided in S3 Movie. All codes and data can be found at https://zenodo.org/doi/10.5281/zenodo.10865520. RD, reaction–diffusion.

Our experiments showed that the AGA treatment stimulates spatiotemporal emergence of ectopic buds. The observed patterning dynamics suggest that the AGA treatment can stimulate new instabilities and potentiate the chick skins to initiate a new developmental program until the skins reach a new equilibrium. The first patterning cues act on a larger scale to set up primary feather buds followed by patterning at smaller scales. Turing’s two-species RD system has successfully explained how stationary patterns can form from an initially homogeneous environment (Fig 13A). However, pattern formation in biological systems often evolves from one pattern (non-homogeneous state) into another. Therefore, the classical Turing model has encountered challenges in explaining more complicated pattern formation processes. To overcome these challenges, Nagorcka and Mooney developed a Turing-type model and applied it to successfully explain the pattern formation of hair follicles [56]. Importantly, the only restriction in their model is that no patterning occurs in the prepatterned region, and there is no asymmetry added in the model. Taking inspiration from their work, we use a rectangular domain (specifically, the simulation domain is [0,15]×[0,25]), with periodic boundary conditions top and bottom and zero-flux conditions left and right. Thus, the rectangle represents a small patch of a much larger domain.

To produce the spots in the order as numbered in Fig 10B, we specify 5 sets of 2 coupled diffusing morphogen species, (*u_i_, v_i_*) *i* = 1,2,3,4,5 that can interact in the same way (Fig 13B). Critically, although all species can diffuse throughout the space, interactions are only able to occur in regions that are not already patterned. Namely, at each stage of pattern formation, the tissue through which the morphogens are moving differentiates and does not allow further interactions to occur, again an idea suggested by Nagorcka and Mooney [56]. Specifically, we assume that the tissue undergoes a mechanical change, supported by the observed differences in LY dye transfer between the bud and the interbud regions (Fig 7) and the reported change in epithelial cell shape in response to local tissue contractility [13], thus, making the morphogens diffuse slower through the tissue.

Explicitly, the equations underlying the simulations are the following:

$$\frac{\partial u_{i}}{\partial t}=D_{u_{i}}\nabla^{2}u_{i}+(0.3-u_{i}+u_{i}^{2}v_{i})\times S_{i}(x,y),$$

$$\frac{\partial v_{i}}{\partial t}=D_{v_{i}}\nabla^{2}v_{i}+(0.8-u_{i}^{2}v_{i})\times S_{i}(x,y),$$

where the kinetics, known generally as Schnakenberg kinetics [57], are fairly arbitrary and are an example of Turing kinetics [1]. Specifically, when the parameters are chosen appropriately, the system will undergo a spontaneous symmetry breaking and produce stationary spatially heterogeneous concentrations of morphogens. These kinetics in particular are well-known for producing simple spot patterns. The $D_{u_{i}}$ and $D_{v_{i}}$ are positive constants, which measure the rate of diffusion. Finally, the *S_i_*(*x,y*) function is a piecewise function that only activates the kinetics in specific regions, *Ω_i_*,

$$S_{i}(x,y)=\left\{ \begin{array}{c} 1\;if(x,y)\in \Omega_{i},\\ 0\;otherwise, \end{array}\right.$$

where *Ω_i_* is to be specified in terms of regions not already patterned. A schematic of how these 5 patterns are formed is shown in the top row of Fig 13B. We start with kinetics that are active everywhere. This system produces the main large spot structure (top left image). Once the first pattern has reached stability, the second Turing system is activated. Critically, although the second set of morphogens is allowed to diffuse everywhere, the reactions are only allowed to occur in the regions where previous activator condensations are not formed. This is shown in Fig 13B. Namely, the second patterning domain is the white region illustrated in the second-from-the-left bottom image. Black regions illustrate where the morphogens can diffuse, but not react. Simulating the system on this domain produces the top second-from-the-left image. This process is repeated until the end of the fifth simulation. In each case of Fig 13B, the bottom image illustrates the morphogenetic field, while the corresponding top image illustrates the final pattern that emerges on the given field. Note that spots 3, 4, and 5 require the space to be much more restricted. Additionally, the only restriction in our model is that no patterning occurs in the prepatterned region, and there is no asymmetry added. Therefore, under different initial conditions a top-bottom flipped version of the simulation may occur, namely the blue spot would appear above the green circle and the side spots would equally be reflected below the green spot. Critically, one of these 2 solutions must occur because this is the minimal energy mode for the pattern wavelength. Namely, the spots cannot be packed closer, and if there was any further space a new spot would appear. Thus, although a top-bottom asymmetry can be easily created, either symmetry is possible. In our simulations, the anterior-posterior axis was defined after the simulation. Fixing the direction of this axis can easily be achieved by applying a morphogen gradient to the green circles, which in turn, would become ovals elongated in the region we did not want to pattern. However, this would require an unverified assumption and, since our theory is phenomenological, we choose to focus on the minimal number of assumptions required to generate a proof-of-concept pattern.

Due to the consequential nature of the bud formation, we can perturb the order of bud development. Specifically in Fig 13C, we simulate the system again with the same parameter values and the influence of buds 1, 3, 4, and 5 are the same in terms of their spatial restriction. However, we have removed the influence of the second bud (blue bud in Fig 13B). This causes the pattern positioning to appear in unexpected places. This result indicates that the ectopic buds in the interbud region are required to break the anterior-to-posterior positional symmetry of the subsequent patterning waves. Notably, these simulations are solely dependent on coupled activator-inhibitor species from an initially homogenous patterning field. Thus, these simulations may not be able to fully recapitulate the patterning processes in developing embryos, namely the changing tissue landscape. From experimental observations, we notice that buds 3, 4, and 5 seem to be confined to the crescent-shaped area and possibly be independent of the placement of bud 2. Therefore in Fig 13D, we simplify the idea of the consecutive simulations by placing a horseshoe region around the first bud to mimic the observed crescent-shaped area around the tissue invagination/evagination site. This specified region is independent of bud 2 and only allows the buds to form in this area. Specifically, the bottom pattern forming field row of Fig 13D illustrates where buds 3, 4, and 5 can appear. In this simulation, we showed that local tissue heterogeneity can complement chemical-based Turing’s patterning mechanism to diversify the final patterning outcome. The technical details of our simulations are described in S1 Appendix. The next challenge will be to see if we can relate the morphogens and kinetic interactions to those chemical signals and interactions present in chick feather patterning.

## Discussion

Studies have demonstrated how Turing’s chemical RD model can be applied to biological systems where morphogen gradient-dependent changes in local cells alter tissue mechanics and generate a global Eda patterning wave that establishes periodic feather bud patterns [2,9,10,15]. Additionally, the zebrafish model showed that cell–cell contact through GJs at cell protrusions can modulate skin pigmentation in a Turing-like manner [22]. In the Japanese quail embryonic skin, GJs has been shown to be involved in longitudinal color stripe patterning across the body [44], and GJA5 (connexin 40) is involved in stripe and spot formation of intra-feather pigment patterning [58]. Unlike the protein-based morphogens that usually diffuse a short distance, gap junctions can communicate over a long range. Thus, gap junctions serve as good candidates to mediate or modulate the long-range inhibitory signal described in Turing’s RD model. How gap junctions may regulate feather bud patterning is still unknown. In this study, we demonstrate that the expression of GJ isoforms, connexins, and GJIC are highly dynamic and strongly associated with feather morphogenesis, with varied functions at different stages. We further show that GJIC inhibition in the skin explant system by AGA results in uncoupling neighboring cells and allows sequential development of ectopic feather buds at new tissue locations across the embryonic chicken skin. Although the specific molecules going through the gap junction are not fully resolved in this work, our results suggest that GJIC can function as a long-range inhibitory signal during periodic feather patterning. The temporal development of ectopic feather buds can be described by the sequential application of Turing systems that present periodic patterns, as shown in our mathematical model. This work is not in conflict with our original findings that FGF and BMP work as diffusible activators and inhibitors for periodic feather patterning [15] as the potential interplay between different types of morphogenetic signals has not been fully resolved. Yet, it reveals an additional layer of GJIC-dependent inhibition in the developing skin. We think the emergence of a new bud is dependent on the net result of interactions between different Turing activator and inhibitor activities, regardless of the forms or sources of those activators/inhibitors.

### Dynamic expression of gap junctions in the developing chicken skin

The permeability of GJs is greatly affected by the composition of GJ isoforms, and different compositions of GJs have their preferred conformation, charge, and substrate size limits [35,59,60]. We show 7 different connexin isoforms exhibiting distinct and overlapping temporal and spatial patterns during early feather morphogenesis (summarized in Fig 5), suggesting that GJs can possibly be formed by not only a single GJ isoform but various combinations of GJ isoforms. The complexity of GJ isoform expression in the chicken skin increases as the body grows, and this would allow GJs to modulate more complex cell type-specific behaviors and/or tissue landscapes during development. Importantly, GJIC can also be modulated by protein kinase-mediated posttranslational modifications, e.g., phosphorylation [61]. These modifications can rapidly affect the assembly or disassembly of GJs at the plasma membrane. Together with the short half-life of GJ isoforms, around 1 to 5 h, these modifications in different types of tissues [62] allow GJs to regulate a dynamic patterning landscape, such as a developing feather field, with messages that can reach long length scales far and fast. Additionally, many of the protein kinases capable of influencing GJIC have known roles in periodic patterning and can be regulated by protein-based morphogens [7,63,64]. This raises an interesting possibility that short-range protein-based morphogens and potentially long-range signals mediated by GJs co-function as signaling networks during feather patterning.

We showed that inhibition of GJIC by AGA and its derivative, CBX, can induce ectopic feather buds. This result suggests the existence of inhibitory factor(s) passing through GJs, and this regulation is required to establish correct boundaries and domains during periodic feather patterning. AGA and CBX are more specific and less toxic compared to other GJIC inhibitor types, such as lipophilic molecules (halothane and octanol) and acidifying agents (doxylstearic acids) [47,50,65]. AGA was proposed to inhibit GJIC by intercalating into the plasma membrane and binding to GJ hemichannels, leading to the closure of GJ channels through conformational changes [47]. CBX was shown to alter arrangements of GJ hemichannels possibly through a direct binding [50]. Notably, we showed in this study that AGA treatment also results in reduced Cx43 protein levels (Fig 12A). Therefore, the ability of Cx43 to act as an adhesion molecule might also contribute to boundary formation and contact inhibition. Because the Cx43 expression and LY dye transfer patterns are similar during early feather patterning, we sought to investigate if specific Cx43 inhibition would lead to a similar phenotype as with AGA treatment. Unfortunately, our attempts to use the specific Cx43 channel blocker, the mimetic peptide GAP27, and siRNA were not successful, likely due to poor tissue permeability and/or short half-life in the skin explant system and embryos. Additionally, our previous study showed that lentiviral-mediated Cx43 down-regulation resulted in suppression of feather bud elongation [38]. The lentivirus was injected into embryos at early developmental stages (H&H stages 14 and 17) before feather field formation, and, in the current study, we showed that Cx43 is expressed in the pteric regions at H&H stage 28 (Fig 3). Lentiviral-mediated Cx43 suppression at the early stage might prevent feather field formation, thus blocking future ectopic feathers from forming at a later stage. Interestingly, our results showed that *Cx30 RNA* expression was elevated following the AGA treatment (Fig 12A). Whether the effect of AGA is mediated by Cx43, Cx30, other GJ isoforms, or a combination, will be studied further.

### Roles of Cx30 during the formation of the primary buds

In developing chicken skin, *Cx30* is one of the earliest detectable GJ isoforms, as demonstrated by WM-ISH during feather patterning (Fig 2). We observed that the treatment with *Cx30* siRNA and/or RCAS-Cx30^ΔC^ viruses resulted in inhibition of feather bud formation and/or an increase in Vimentin expression in the epithelium (Fig 6). These results suggest that Cx30 may participate in feather bud development and the specification of epithelial cell fate. While the underlying mechanisms will need to be explored further, we can gain some insights into the cellular and molecular functions of Cx30 from studies conducted in humans and mice. Connexins have been linked to many types of human diseases and congenital skin diseases and they can play diverse functions in different contexts [66–69]. Cx30 (encoded by the *GJB6* gene) mutations in humans account for hearing loss and hidrotic ectodermal dysplasia (Clouston syndrome), which is characterized by severe hair loss, nail hypotrophy, and palmoplantar hyperkeratosis. Human Cx30 (a.a. 1–261) shares 73% of amino acid sequence identity with chick Cx30 (a.a. 1–263). A previous study using human Cx30 (a.a. 1–215), devoid of the COOH-terminal region, showed that this Cx30 mutant mis-localized intracellularly and could not form GJ plaques on the plasma membrane in the HSC-4 human head-and-neck cancer cell line [70]. Cx30 A88V homozygous mutant mouse exhibited leaky Cx30 hemichannels with increased ATP release and calcium ion influx in primary keratinocytes [71], suggesting that GJs formed by wild-type Cx30 could mediate the transfer of ATP and calcium ions. Interestingly, the Cx30 knockout mice created by deletion of the coding region exhibited abnormal endocochlear potential and degeneration of auditory hair cells but, in contrast to humans, showed normal skin and hair development [72]. This is likely due to redundant Cx regulatory networks operating in mouse. A previous study showed that hair, feather, and scale are homologous and share common molecular and micro-anatomical characteristics during placode development (H&H stage 28 to 31 in the chick embryo; the stages overlapped with our studies) [73]. Additionally, as an example, early studies showed that β-catenin can initiate both hair follicle and feather bud development [8,74]. The early expression of Cx30 during placode development, high Cx30 amino sequence identity between chicken and human, and the similarity of the inhibition of feather bud development in Cx30-perturbed chick embryos and the hair loss phenotype in human Clouston syndrome suggest that there is a potential to further develop embryonic chicken skin as a model system to study the human disease.

Additionally, it would be of particular interest to know if Cx30 and Cx43 can co-regulate the earliest stage of feather patterning, because these 2 connexin isoforms both showed early gene expression and the knock-down experiments performed with RCAS-shCx43 in the previous study [38] and siRNA and RCAS-Cx30^ΔC^ viruses in the current work exhibited a similar inhibitory phenotype. It is also intriguing to further study the connections between β-catenin, Cx30, and Cx43 in the regulation of bud size and differentiation during early feather patterning given the interesting dot and circle patterns observed in our studies (Fig 2).

### Gap junction communication may contribute to the Turing long-distance lateral inhibition during feather pattern formation

In this study, we surprisingly uncovered the fact that inhibition of GJIC allows the sequential emergence of new bud formation in ex vivo cultured skin explants while maintaining the hexagonal patterning of primary feather buds (S3 Fig). This observation suggests that the primary feather array (spot 1 in Fig 10) is established by the sum of Turing activators/inhibitors, whether they are physical cues or morphogens [9,75] available at the time of formation. In our experimental conditions that remove the inhibitory activity contributed by gap junctions, new buds temporally emerge in specific locations, reflecting the dynamic changes of the morphogenetic competent field once patterning starts. The ectopic buds first emerge in the interbud region with maximal distance to adjacent primary buds (Fig 9A and spot 2 in Fig 10). This suggests that GJIC inhibition may restrict the distance of diffusion of inhibitory factors originated from the primary feather buds. Hence, it lowers the threshold for bud growth and allows the emergence of ectopic buds in the initially suppressive interbud region. Second, we found that GJIC inhibition results in increased cell proliferation in the anterior bud base (Fig 11D) and the contribution of cell mass from the primary bud to the ectopic bud (Fig 11E and spot 3 in Fig 10). These results indicate that GJIC inhibition could also allow for the emergence of ectopic feather buds through enhancing the activator signal, hence the cell density [7,10]. Additionally, the increase in cell mass (Fig 11D) and the activation of β-catenin (Fig 12C) may collaboratively contribute to the modification of tissue mechanics, resulting in the cell shape change (Fig 12B) and bulging out tissue structure as suggested by a previous study [13]. Of note, we found that new buds at spots 4 to 5 (Fig 10) are localized in a more confined area around the base of the primary feather buds. We speculate that the intrinsic higher cell mass at this region [76] (Fig 11A and 11C, triangle-headed arrows) provides extra activation cues for the cells in this area to assemble feather buds. Taken together, a hidden layer of Turing-type patterning possibilities is revealed by the removal of gap junctional communications. In our living experimental system, we can explain the results by stating that the threshold for bud formation is reached by the summed activators and inhibitors in that particular space and time.

We then provide a proof-of-concept mathematical model to account for the observed patterning behavior. The classical Turing theory is generally applied to 2 morphogens acting upon homogeneous environments, which have simple n-dimensional shapes (e.g., lines in 1 dimension, rectangles in 2 dimensions, cuboids in 3 dimensions) with simple boundary conditions. However, even Turing noted that biology presents greater challenges since “most of an organism, most of the time is developing from one pattern into another, rather than from homogeneity into a pattern” [1]. Critically, a developing embryo is a growing 3D morphogenetic field. As such, tissue curvature and mechanics in developing embryos add extra layers of complexity of engaging diffusive factors in the patterning processes. In our reduced skin explant system and 2D simulations, we minimized these concerns and showed that the Turing-like patterns could emerge under our experimental conditions. Furthermore, our observations of the temporal appearance of stabilized Turing-type patterns in gradually restricted regions following each patterning cue suggest the possibility that earlier patterning events could progressively modulate the Turing patterning landscape. Moreover, the self-organizing property of feather buds not only sets up the size and spacing of feather primordia, but also polarity. Preferential accumulation of cells at specific directions or locations, e.g., the clustered progenitor cells around the anterior base of primary feather buds, could reflect tissue mechanics, direction of stress, or flow of morphogenetic cues, and provide activator signal(s) to further modify the patterning landscape [77]. The observed order of temporal patterning is revealed only when we treat skin explants with GJIC inhibitors, thus presumably removing the lateral inhibitors generated by primary feather buds. The appearance of ectopic buds in the interbud regions (spot 2) further showed the possibility of the existence of long-range inhibitor(s) mediated by gap junctions. This is beyond the traditional view of protein-based morphogens which can only diffuse over a much shorter range in a complex tissue system. We suspect the possible long-range inhibitors mediated by gap junctions include the following candidates: ions, e.g., calcium and potassium ions, bioelectricity, second messengers, and tissue mechanics. Notably, in recent years, the identity of factors that are able to generate Turing-like patterns has been greatly expanded in biological systems [2,10]. It would be exciting to further decipher how biochemical, mechanical, and bioelectrical signals interplay with dynamic cellular behaviors and tissue development, e.g., cell proliferation, migration, differentiation, and body growth, to reach a new stable state in these fast-changing patterning processes.

Research on periodic feather pattern formation has mostly focused on patterning of primary feather follicles. It is still largely unknown how the young and adult feather types are specified and spatially positioned during skin development. Newborn baby chicks are covered by fluffy down feathers which are gradually replaced by adult feather types by weeks 6 to 12. There are 4 main forms of feathers that cover the body of the chick, including contour feathers, semiplumes, filoplumes, and down feathers. In this work, we did not specify the identity of these emerging buds because skin explants are not suitable for long-term culture. It would be interesting to further investigate how these ectopic buds are correlated with mature feather types in terms of their identity, size, density, and spatial localizations. Similar consecutive patterning mechanisms have been observed in mammalian hair such as wool secondary follicle formation [78,79] and fetal hair development [56]. We speculate that GJ-associated patterning mechanisms could be applied to a broader range of living systems during development.

Finally, we qualitatively compared the expression of connexins in developing chicken embryo skin included in this study with what was previously reported [38]. The major aim of this rough comparison is to highlight the connexins expressed in the skin that were not included in this study, and less importantly to provide updated gene names and aliases for each connexin to cross-validate their expression. Note that these 2 studies utilized different experimental methods (WM-ISH versus RNA-seq). The RPKM (reads per kilobase million) numbers of the RNA-seq results obtained from the GEO dataset (GSE86251) deposited by the previous study were summarized in S2 Table. The qualitative comparisons were shown in S3 Table. Key observations, excluding the results showing lower expression levels, are: (i) GJB3 and GJC2 are not included in this study but showed moderate and tissue-specific expression in the epithelium and mesenchyme, respectively, in the RNA-seq results; (ii) GJA4 was not detected in this study but showed moderate expression in the mesenchyme and low expression in the epithelium; (iii) GJA1, GJA5, and GJB6 showed moderate to high expressions in both studies; (iv) GJB2 was not included in both studies; and (v) most of the connexins showing low expression in one study also exhibited low or no expression in another study. According to these observations, we suggest that future research on early feather patterning should examine the expression and functions of GJB3, GJC2, and GJB2 in more detail.

Overall, the findings shown in this work suggest that gap junctions play a critical role in feather pattern formation. While there are limitations, as mentioned in the discussion above, this study offers new insights into the mechanisms that underlie the emergence of complex spatial patterns in developing skin and in biological systems beyond the skin. A fascinating topic for future investigation is to find out what signaling can be mediated by gap junction communications in periodic patterning, how far and how fast.

## Materials and methods

### Embryos

Fertilized White Leghorn chicken eggs were obtained from Charles River (specific pathogen-free) or the local farm (AA Laboratories, Westminster, CA). The eggs were incubated at 38.5°C in a humidified chamber and staged according to Hamburger and Hamilton (1951).

### Culturing chicken embryonic fibroblasts

Specific pathogen-free St31 chicken embryos were collected and decapitated. Internal organs and limbs were removed with sterile forceps. The embryos were macerated with sterile scissors and then dissociated with 0.05% trypsin with EDTA. The disassociated cells were filtered through 70 μm nylon filter and plated on 100 mm culture dishes in DMEM/high glucose supplemented with 10% fetal calf serum (FCS) and 2% chicken serum.

### Production of RCAS retroviruses

RCAS-Cx30^ΔC^ or the backbone plasmids were transfected into freshly prepared chicken embryonic fibroblasts or DF-1 cells by the treatment of 250 mM CaCl2 and 2X HeBS for 4 h at 37°C. Cells were then briefly treated with 15% sterile glycerol in phosphate-buffered saline (PBS) and washed with PBS. Then, they were incubated at 37°C overnight in DMEM with 10% FCS and 2% CS. The day after transfection, culture media was replaced with 10 ml DMEM with 1% FCS and 0.2% CS. The media was filtered and collected for 3 consecutive days and then pooled together and centrifuged at 4°C at 20,000 rpm for 3 h. One tenth of the original culture media containing viral particles were gently shaken at 4°C overnight. The media were then aliquoted in 100 μl and stored at −80°C until use.

### Injection of RCAS viruses

The aliquoted virus was thawed briefly at 37°C. Volumes of about 5 to 10 μl were injected into the amniotic cavity of chicken embryos at H&H stage 16/17 or 18.

### Skin explant culture

The dorsal skins of chicken embryos at St28 or St34 were dissected in Hank’s buffered saline solution under a dissection microscope and then placed onto culture inserts in 6-well culture plates (Falcon, 08-771-15). Skin explants were cultured in DMEM/high glucose supplemented with 10% FBS in a humidified chamber maintained at 37°C at an atmosphere of 5% CO2 and 95% air. The media were replenished every other day.

### GJIC inhibitor treatment

The reversible small molecule inhibitors AGA (Sigma-Aldrich, G8503) and its analog, glycyrrhizic acid (Sigma-Aldrich, 50531), were dissolved in DMSO at a concentration of 50 mM. The reversible AGA-derivative, carbenoxolone (CBX; Sigma-Aldrich, C4790) was dissolved in DPBS at a concentration of 100 mM. The concentrated stock solutions were diluted in DMEM with 10% FBS and 2% CS to make 100 μm working solutions right before the experiments. The culture media were replenished every other day.

### Scrape-loaded Lucifer yellow dye transfer assay

Lucifer yellow CH dipotassium salt (Sigma, L0144) and rhodamin dextran (Invitrogen, D1824) were dissolved in distilled water to make a 1% stock solution. The working solution was made by mixing 100 μl LY, 100 μl rhodamine dextran stock solution, and 300 μl DPBS, and 20 to 40 μl of working solution was applied to each freshly prepared dorsal skin before scraping, and the LY was allowed to transfer for 8 min. Then, the skins were briefly washed with PBS and fixed with 4% paraformaldehyde (PFA) for 20 min at room temperature (RT). The samples were imaged by either Zeiss 510 confocal microscope or an epifluorescent microscope.

### Probe making for WM-ISH

The coding sequences of connexins were submitted to Primer3 for primer design. The promoter sequence for T7 RNA polymerase was added on the 5′ end of antisense primer. The primer sequences used in this study were listed in S1 Table. The PCR products at the size of around 500 bp were amplified from the mixture of St31 and St34 chicken cDNAs. The PCR products were then sequenced to verify the identity and transcribed with T7 RNA polymerases to obtain the antisense probes labeled with digoxigenin. The probes for *β-catenin* and *shh* were described previously [7].

### siRNA design

The mRNA coding sequence of *Cx30* was submitted to Ambion siRNA Target Finder to generate candidate siRNA sequences. The final siRNA sequence was selected according to the following criteria: (i) the sequences having 4 or more Gs in a row were avoided; (ii) the sequences of 30% to 50% GC were submitted to the BLAST to ensure that they only will anneal to their intended cognate sequence; and (iii) no other similarities in the chick genome. The randomized sequence with the same ATCG composition was used as the control. The siRNAs were synthesized by Thermo Scientific. The oligonucleotides for *Cx30* siRNA and the randomized control are listed in the S1 Table.

### In ovo siRNA electroporation

The siRNAs at the concentration of 100 μm were stored as 10 μl aliquots at −80°C. Immediately before use, a small drop of FastGreen was added to the aliquots, and the siRNAs were used at the highest concentration. The siRNAs were injected into the subepidermal layer of embryos at H&H stage 26 with 1 to 2 μl. The electrodes were put on the flank of embryos. The siRNAs were transferred to cells using electroporation at 16 V (the actual output is 12 V), 50 ms 3 times.

### RT-qPCR for skin explant experiments

Individually collected skin samples were pestle-homogenized in microcentrifuge tubes. RNA extraction was performed with the TRIzol reagent, followed by cDNA synthesis using the SuperScript III First-Strand Synthesis kit. Two micrograms of each RNA sample were used for the cDNA synthesis. Primers used for qPCR are listed in S1 Table. The qPCR was performed using the Applied Biosystems QuantStudio 3 system and analyzed by the Design & Analysis Software version 2.7.0.

### Construction of RCAS plasmids

The COOH-terminal Flag-tagged truncated Cx30 (a.a. 1–214) coding sequence was amplified by the primers listed in S1 Table with the PCR reactions and then sequenced to make sure the accuracy of the sequence. Then, the target sequence was cloned into the RCAS-2A-mcherry plasmid by ligation. The resulting plasmids were transfected into the One Shot OmniMAX 2-T1R Chemically Competent cells (Invitrogen, C8540-03) by heat shock. The cells containing the desired plasmids were amplified by broth culture and the plasmids were prepared by maxiprep kit (Qiagen, 12663). The plasmids were stored at −20°C until use.

### Sample processing

Embryos for WM-ISH were collected in DEPC-treated PBS containing 0.1% Tween-20 (DEPC-PBT) and then fixed with 4% PFA in DEPC-treated water at 4°C overnight. Then, samples were dehydrated through a methanol gradient and stored at –20°C. Samples for tissue sectioning were fixed with 4% PFA and then dehydrated through an ethanol gradient. Then, samples were treated with xylene twice and embedded in paraffin. Thickness of tissue sections: 14 μm for WM-ISH samples (except for Cx40, 20 μm) and 7 μm for immunofluorescence and HE staining.

### Whole-mount in situ hybridization (WM-ISH)

Dehydrated embryos were gradually rehydrated into DEPC-PBT and then bleached with 6% H_2_O_2_ in DEPC-PBT followed by treatment with 20 μg/ml Proteinase K in DEPC-PBT. Samples were post-fixed in freshly prepared 0.25% glutaraldehyde/4% PFA solution and then pre-blocked in the hybridization buffer. Digoxigenin-labeled probes were applied to the samples at 65°C overnight. Samples were then washed thoroughly with 2× and 0.2× sodium chloride-sodium phosphate-EDTA Buffer (SSC buffer) for 4 h. Then, samples were blocked by 20% heat-inactivated goat serum in DEPC-PBT for 2 h and treated with pre-absorbed anti-digoxigenin antibody conjugated with alkaline phosphatase at 4°C overnight. Samples were then washed in DEPC-PBT containing 1% levamisole for 3 h followed by washes in NTMT (100 mM NaCl, 100 mM Tris-HCl, 50 mM MgCl_2_, and 0.1% Tween-20) containing 1% levamisole for 2 h. Colors were developed with NBT/BCIP, and the reactions were stopped by PBS.

### Immunofluorescence and confocal microscopy

Tissue sections were rehydrated through an ethanol gradient. Antigen retrieval was performed with 10 mM citric acid buffer (pH 6.0) at 95°C for 30 min. After samples were cooled down to RT, they were blocked at RT for 2 h followed by incubation with primary antibodies at 4°C overnight. Then, samples were incubated with secondary antibodies conjugated with Alexa Fluor 488 (green) or Alexa Fluor 594 (red) at RT for 2 h. After washing, samples were mounted with Vectashield anti-fade medium with DAPI (H-1200, Vector Laboratories). Images were acquired by a Zeiss LSM510 confocal microscope equipped with LSM 510 Version 4.2 SP1 acquisition software (Carl Zeiss). Antibodies: Cx43 (Santa Cruz Biotechnology, sc-9059); E-Cadherin [41]; pY489-β-catenin (Developmental Studies Hybridoma Bank).

### Immunohistochemistry (IHC)

Tissue sections were briefly heated at 65°C for 5 min and deparaffinized in xylene, followed by rehydration through an ethanol gradient. Then, 6% H_2_O_2_ in methanol was used to block the endogenous peroxidase. Antigen retrieval was performed with 10 mM citric acid buffer (pH 6.0) at 95°C for 30 min. Sections were allowed to cool down for 30 min at RT. Then, the sections were pre-blocked by the zeller’s solution for 2 h at RT. Primary antibodies were diluted in the zeller’s solution and incubated with tissue sections at 4°C overnight. Then, biotin-linked secondary antibodies were applied at 4°C overnight. The next day, streptavidin-horseradish peroxidase was labeled for 2 h at RT. Colors were developed with 3,3′-diaminobenzidine (DAB) (Vector, SK-4100) for 5 to 15 min. Antibodies: ASLV viral capsid protein p27 (RCAS; Charles River Laboratories, p27); Vimentin (Developmental Studies Hybridoma Bank, H5).

### Whole-mount BrdU staining

BrdU stock solution was made by dissolving BrdU powder in Hank’s Buffered Salt Solution (HBSS) at the concentration of 1.5 mg/ml and was stored at −20°C. Skin explants were labeled with 150 μg/ml BrdU for 4 h and then fixed with 100% methanol for 2 h. Then, skin explants were treated with 10% H_2_O_2_ in 1:4 DMSO: methanol for 2 h to block the endogenous peroxidase activity. Samples were then treated with 20 μg/ml Proteinase K in PBT at RT for 7 min. Then, samples were briefly washed and fixed with 4% paraformaldehyde/0.1% glutaraldehyde in PBS at RT for 20 min followed by treatment with 2N HCl in PBT for 1 h. Samples were then treated with 0.1 M sodium borate buffer, pH = 8.5 and labeled with the mouse anti-BrdU antibody (Millipore, MAB3424) at 1:1,000 dilution followed by the incubation with biotin-linked anti-mouse antibody and streptavidin-horseradish peroxidase. Colors were developed with DAB (Vector, SK-4100).

### DiI labeling

The Vybrant DiI cell-labeling solution (Invitrogen, V22885) at the concentration of 25 μm was injected into multiple locations on the skin explants that were harvested at H&H stage 34 and then ex vivo cultured for 1 day. Images were taken for 4 days on an epifluorescent microscope.

### Scanning electron microscopy

Freshly isolated chicken dorsal skins were fixed with Karnovsky’s fixative at 4°C overnight and then post-fixed with thio-carbohydrazide followed by fixation with osmium textroxide. Specimens were critically pointed dried and then coated with gold palladium. Images were acquired with a JEOL JSM-6390LV scanning electron microscope at the Doheny Eye Institute.

### Mathematical simulation

Numerical simulations of the RD systems were run using the finite element software COMSOL Multiphysics 5.3 using a Backward Differentiation Formula scheme in time. The first bud simulation was run for 500 time units before the subsequent RD systems were initiated. Each further RD system was run after 1,000 time units. The domain was chosen to have a discretization of 25,000 triangular elements. In each case, representative simulations were rerun with halved mesh sizes to ensure that the observed patterns did not change with discretization. All codes and data can be found at https://zenodo.org/doi/10.5281/zenodo.10865520. Additional technical details were listed in S1 Appendix.

## Supporting information

S1 Fig *Cx43* expression in the chicken embryo at H&H stage 31. *Cx43* RNA was visualized by whole-mount in situ hybridization. A, anterior. P, posterior.
(TIF)

S2 Fig The treatment of AGA effectively suppressed GJIC and induced the formation of ectopic feather buds. (A) Scrape-loaded LY dye transfer assay. H&H stage 34 skins were harvested and treated with AGA or DMSO control. LY and Rho dyes were loaded the next day. Left 2 panels: images were taken by the Zeiss LSM 510 confocal microscope. LY is in green. Rho is in red. A, anterior. P, posterior. Scale bars, 100 μm. The right panel shows the fluorescence intensities of LY and Rho along the indicated yellow lines shown in the left 2 panels. The values of the intensities were obtained using Image J software. Raw data of the measurements is available in S1 Data. (B) Bright-field images showing H&H stage 34 skin explants treated with AGA, glycyrrhizic acid (a non-functional synthetic analog of AGA) or DMSO control for 5 days. A, anterior. P, posterior. Scale bars, 300 μm. (C) Whole-mount in situ hybridization (WM-ISH) of embryonic chicken dorsal skin explants treated with AGA. H&H stage 34 skins were harvested and then ex vivo cultured for 5 days. The probes for in situ hybridization targeted *β-catenin*, the early transcriptional markers of feather primordia formation. A, anterior. P, posterior. Scale bar, 100 μm.
(TIF)

S3 Fig Inhibition of GJIC by AGA stimulates the formation of ectopic feather buds at earlier developmental stages. Bright-field micrographs showing the skin explants harvested from the chick embryos at the pre-placode stage (H&H stage 28) and then treated with AGA (*n* = 10) or DMSO control (*n* = 7) for 5 days. Dashed lines show the hexagonal pattern of the primary feather array. The arrows indicate the ectopic feather buds localized around the base of the primary feather buds. A, anterior. P, posterior. Scale bars, 300 μm.
(TIF)

S4 Fig Quantifications show the spatial heterogeneity of primary and ectopic bud combinations. The analyses were performed on H&H stage 34 skin explants treated with AGA for 5 days. The data were collected from 6 biological replicates, and the data analysis was performed with GraphPad Prism software. Raw data is available in S1 Data. (A) Boxed areas highlight the thoracic-lumbar regions used for data collection and analysis. A, anterior. P, posterior. (B) The box-and-whiskers plot shows the percentage of the indicated bud combinations observed in the middle (mid), right, or left thoracic-lumbar region. The line in the middle of the box indicates the median and the whiskers represent the minimum or the maximum value. (C) The statistical significance of the appearance of the indicated bud combinations in different tissue regions was analyzed with two-way ANOVA and Tukey’s multiple comparisons test. Adjusted *P* value: ns, *P* > 0.1234; *, *P* < 0.0332; **, *P* < 0.0021. (D) The heatmap shows the percentage of observed ectopic buds at individual numbered locations (spots 2, 3, 4, 4’, 5, or 5’), regardless of the bud combinations, in each skin explant sample (#1–6). The mid-region of the third sample was excluded from the analysis because it is too crowded to quantify the number of ectopic buds.
(TIF)

S1 Table Oligonucleotides used in the study. (XLSX)

S2 Table The expressions of gap junction isoforms in dorsal skins of chick embryos at H&H stage 31 and 35. (XLSX)

S3 Table Qualitative comparisons of gap junction isoform expressions as shown in this and the earlier study (GSE86251). (XLSX)

S1 Data Raw data used for quantifications. (XLSX)

S1 Movie Animation of emerging feather primordia. (MP4)

S2 Movie Animation of emerging feather primordia in the absence of spot 2. (MP4)

S3 Movie Animation of emerging feather primordia with the complementation of tissue landscape. (MP4)

S1 Appendix Parameters used in computational simulations. (PDF)

## Abbreviations

AGA: 18 α-glycyrrhetinic acid
EDA: ectodysplasin A
FCS: fetal calf serum
GJ: gap junction
GJIC: gap junctional intercellular communication
HBSS: Hank’s Buffered Salt Solution
HE: hematoxylin and eosin
IHC: immunohistochemistry
LY: lucifer yellow
PBS: phosphate-buffered saline
PFA: paraformaldehyde
RCAS: replication-competent avian sarcoma-leukosis virus long terminal repeat with splice acceptor
RD: reaction–diffusion
RPKM: reads per kilobase million
RT: room temperature
WM-ISH: whole-mount in situ hybridization
