# Supplementary figures and images for: Gap junctions in Turing-type periodic feather pattern formation
Source: PLoS Biol. 2024 May 14;22(5):e3002636. doi: 10.1371/journal.pbio.3002636 (PMC11161087; doi:10.1371/journal.pbio.3002636)

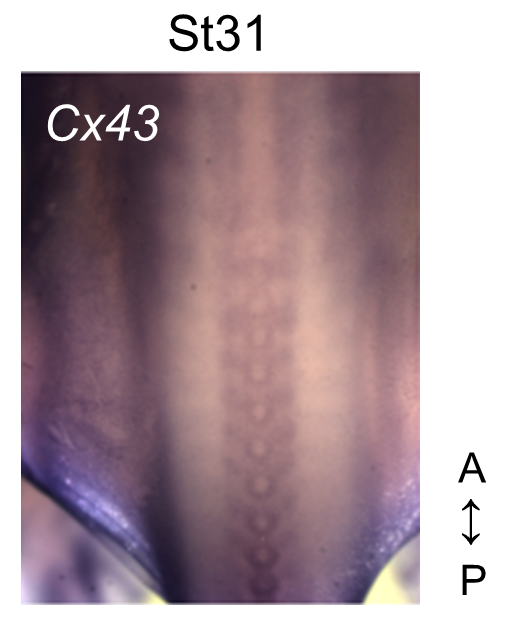

Supplement: S1 Fig — Cx43 RNA was visualized by whole-mount in situ hybridization. A, anterior. P, posterior. (TIF) [file pbio.3002636.s001.TIF]

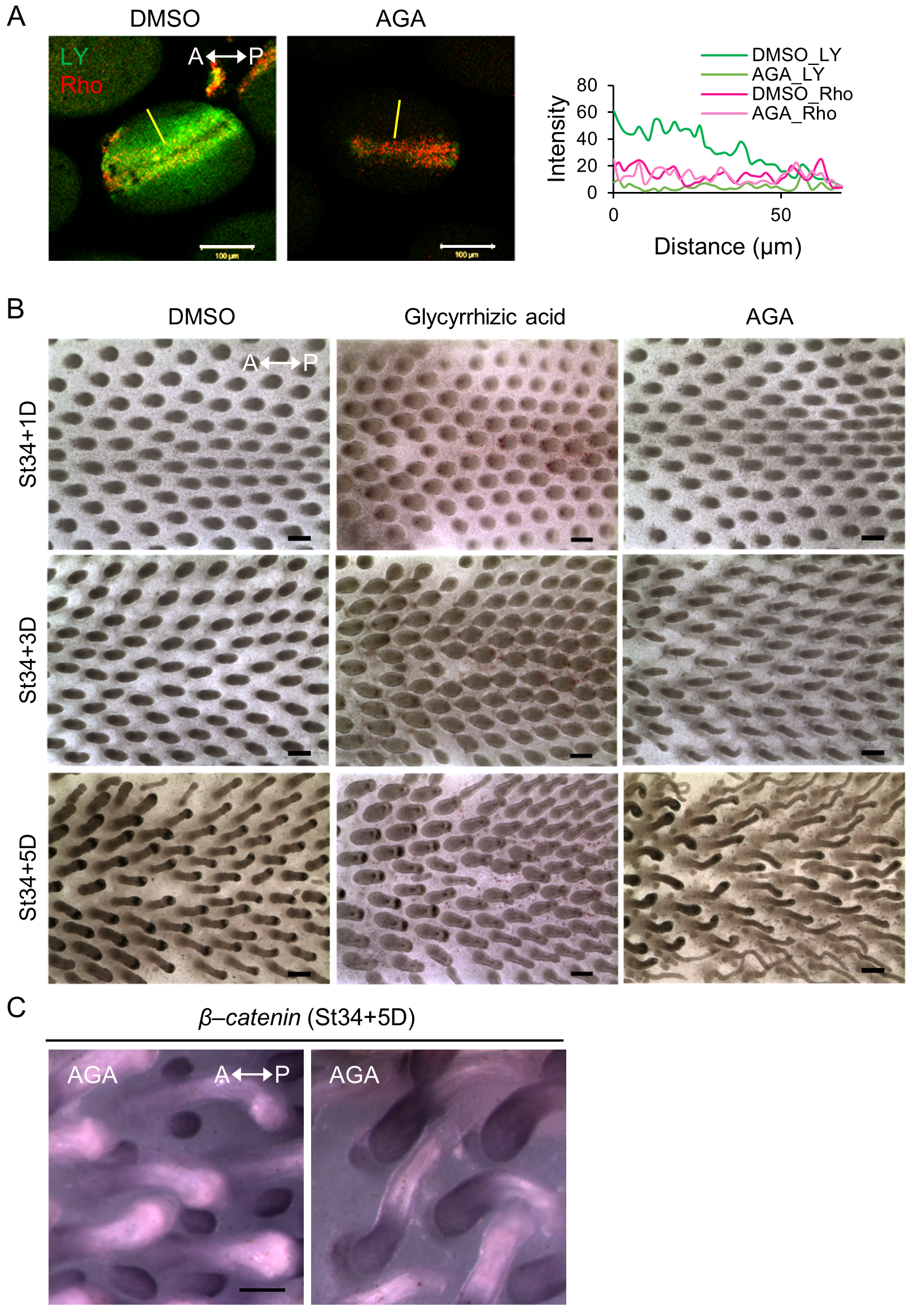

Supplement: S2 Fig — (A) Scrape-loaded LY dye transfer assay. H&H stage 34 skins were harvested and treated with AGA or DMSO control. LY and Rho dyes were loaded the next day. Left 2 panels: images were taken by the Zeiss LSM 510 confocal microscope. LY is in green. Rho is in red. A, anterior. P, posterior. Scale bars, 100 μm. The right panel shows the fluorescence intensities of LY and Rho along the indicated yellow lines shown in the left 2 panels. The values of the intensities were obtained using Image J software. Raw data of the measurements is available in S1 Data. (B) Bright-field images showing H&H stage 34 skin explants treated with AGA, glycyrrhizic acid (a non-functional synthetic analog of AGA) or DMSO control for 5 days. A, anterior. P, posterior. Scale bars, 300 μm. (C) Whole-mount in situ hybridization (WM-ISH) of embryonic chicken dorsal skin explants treated with AGA. H&H stage 34 skins were harvested and then ex vivo cultured for 5 days. The probes for in situ hybridization targeted β-catenin, the early transcriptional markers of feather primordia formation. A, anterior. P, posterior. Scale bar, 100 μm. (TIF) [file pbio.3002636.s002.TIF]

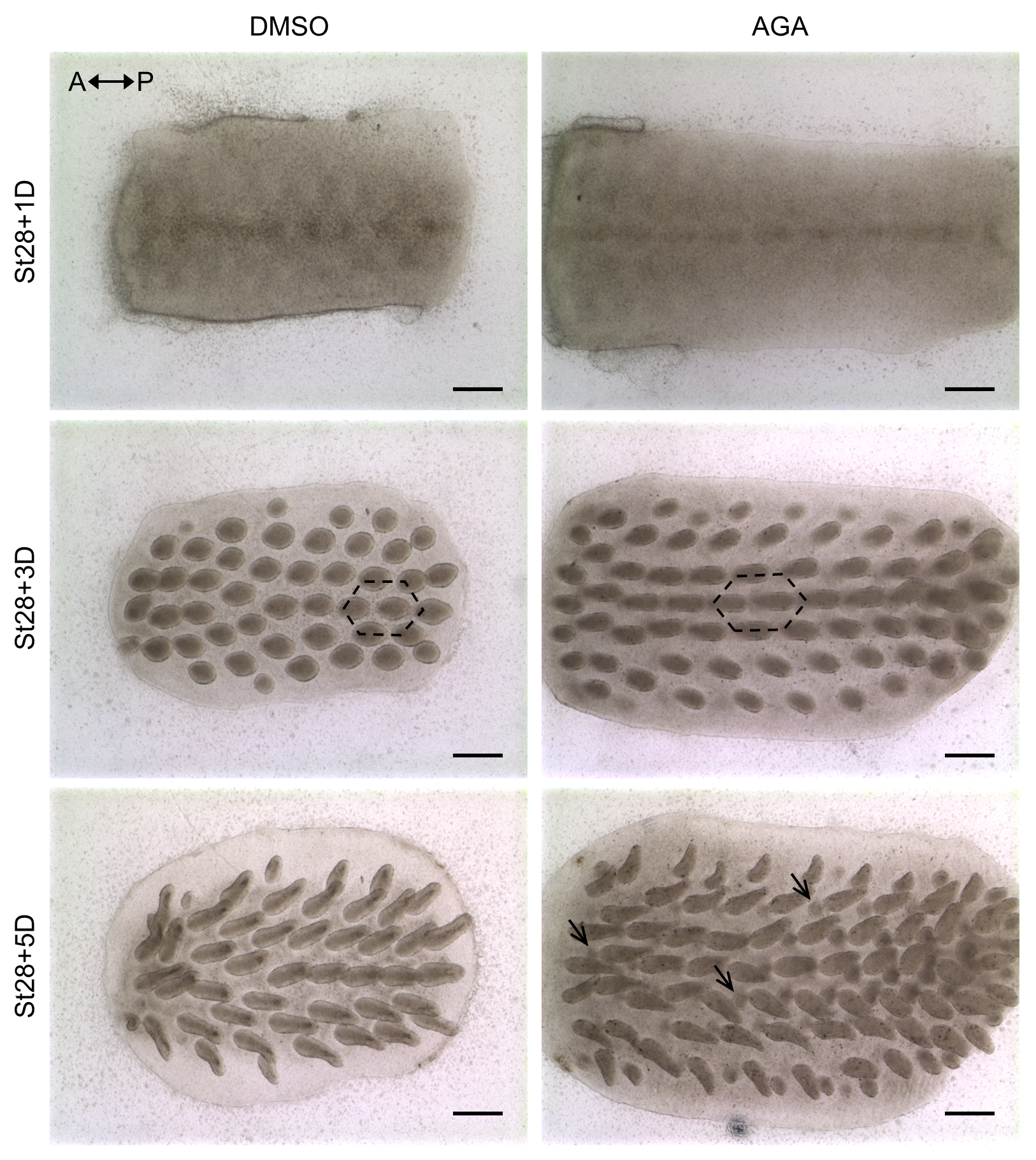

Supplement: S3 Fig — Bright-field micrographs showing the skin explants harvested from the chick embryos at the pre-placode stage (H&H stage 28) and then treated with AGA (n = 10) or DMSO control (n = 7) for 5 days. Dashed lines show the hexagonal pattern of the primary feather array. The arrows indicate the ectopic feather buds localized around the base of the primary feather buds. A, anterior. P, posterior. Scale bars, 300 μm. (TIF) [file pbio.3002636.s003.TIF]

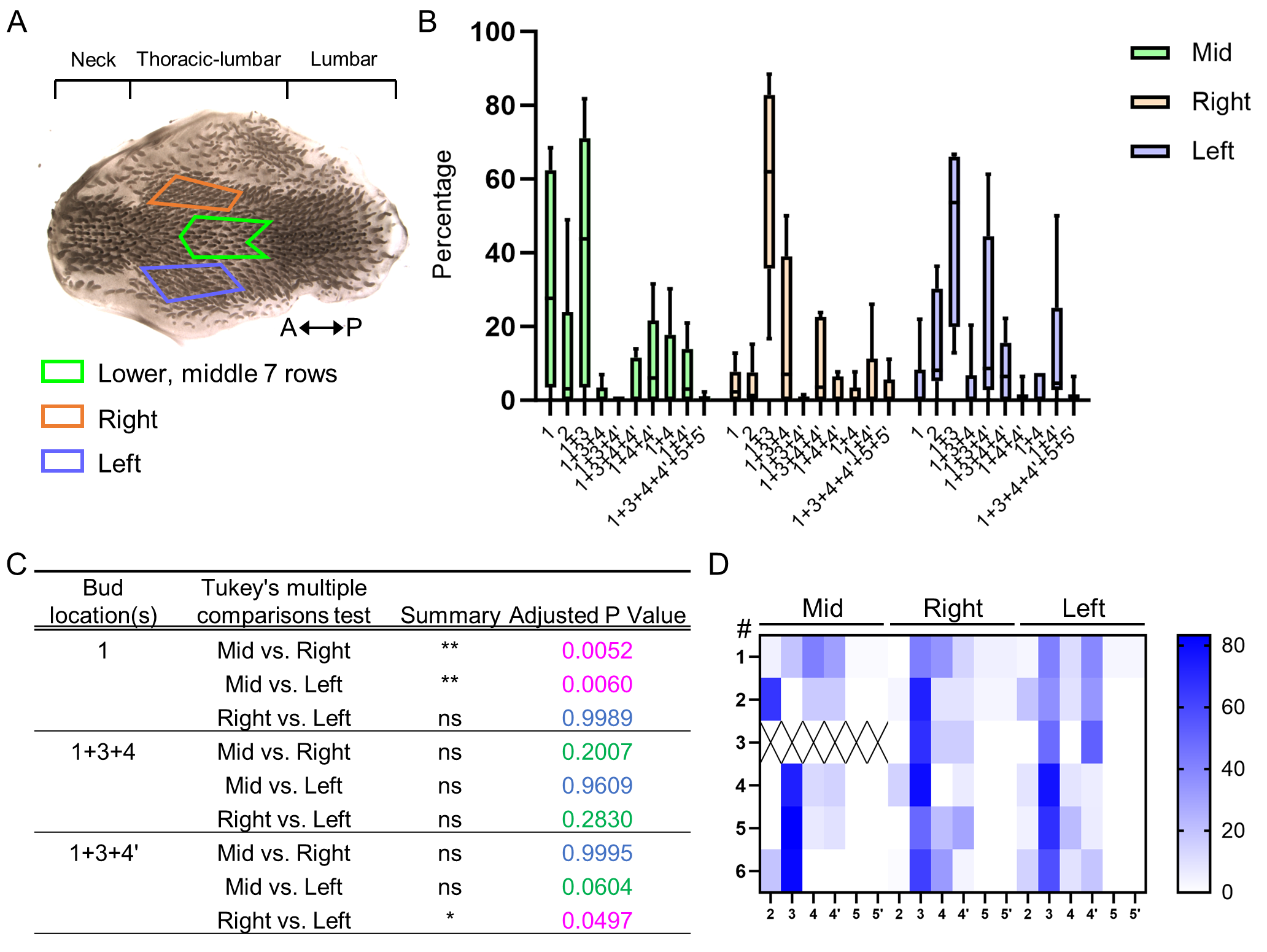

Supplement: S4 Fig — The analyses were performed on H&H stage 34 skin explants treated with AGA for 5 days. The data were collected from 6 biological replicates, and the data analysis was performed with GraphPad Prism software. Raw data is available in S1 Data. (A) Boxed areas highlight the thoracic-lumbar regions used for data collection and analysis. A, anterior. P, posterior. (B) The box-and-whiskers plot shows the percentage of the indicated bud combinations observed in the middle (mid), right, or left thoracic-lumbar region. The line in the middle of the box indicates the median and the whiskers represent the minimum or the maximum value. (C) The statistical significance of the appearance of the indicated bud combinations in different tissue regions was analyzed with two-way ANOVA and Tukey’s multiple comparisons test. Adjusted P value: ns, P > 0.1234; *, P < 0.0332; **, P < 0.0021. (D) The heatmap shows the percentage of observed ectopic buds at individual numbered locations (spots 2, 3, 4, 4’, 5, or 5’), regardless of the bud combinations, in each skin explant sample (#1–6). The mid-region of the third sample was excluded from the analysis because it is too crowded to quantify the number of ectopic buds. (TIF) [file pbio.3002636.s004.TIF]
