# Supplementary material for: Gap junctions in Turing-type periodic feather pattern formation
Source: PLoS Biol. 2024 May 14;22(5):e3002636. doi: 10.1371/journal.pbio.3002636 (PMC11161087; doi:10.1371/journal.pbio.3002636)
Supplement: S1 Appendix — (PDF) [file pbio.3002636.s012.pdf]

S1 Appendix

Technical details for the computational simulations

All codes and data can be found at https://zenodo.org/doi/10.5281/zenodo.10865520. Definitions of the parameters and active regions during each simulation stage. Note the threshold value defines the morphogen region that becomes feather placode.

Since we are dealing with nondimensional parameter values pattern sizes are simply relative to one another. Thus, parameter values were chosen to both produce patterns and so that the final relative sizes of the patterns were consistent with observations.

Figure 13B

Stage	(D_{u_i}, D_{v_i})	Ω_i	Threshold
1	(1,40)	$[0,15] \times [0,25]$,	$u_{Bud} > 1.5$
2	(3/4,30)	$u_{Bud} < 1.5$	$u_1 > 2.6$
3	(2/5,8)	$u_1 < 0.59$	$u_2 > 2.2$
4	(1/10,4)	$u_2 < 0.8$	$u_3 > 2.4$
5	(1/10,4)	$(u_{Bud} > 0.8)(u_1 > 0.9)(u_2 > 0.9)$	$u_4 > 2.55$

Figure 13C

Stage	(D_{u_i}, D_{v_i})	Ω_i	Threshold
1	(1,40)	$[0,15] \times [0,25]$,	$u_{Bud} > 1.5$
3	(2/5,8)	$u_{Bud} < 1.5$	$u_2 > 2.2$
4	(1/10,4)	$u_3 < 0.8$	$u_3 > 2.4$
5	(1/10,4)	$(u_{Bud} > 0.8)(u_1 > 0.9)(u_2 > 0.9)$	$u_4 > 2.55$

Figure 13D

Stage	(D_{u_i}, D_{v_i})	Ω_i	Threshold
1	(1,40)	$[0,15] \times [0,25]$,	$u_{Bud} > 1.5$
2	(3/4,30)	$u_{Bud} < 1.5$	$u_1 > 2.6$
3, 4, 5	(1/30,4/3)	$(u_1 > 0.8)(u_1 < 1.2)((y < 2)(y > -6) + (y < 15)(y > 6) + (y < -11))$	$u_2 > 2.2$

Initial conditions

For the initial bud simulation the initial condition is a spot of activator in the centre,

$I_1(x, y) = \exp(-(x - 7.5)^2 - (y - 12.5)^2)$. Although a specific condition is used, it is a fairly weak requirement because the same pattern will form under any initial conditions due to the wavelengths chosen by the Turing pattern. The only difference will be that the pattern will be translated left, or right, because of

the periodic boundary conditions. Thus, we choose the centered Gaussian to centralize the pattern and, hence, provide simple visual results.

For every other stage the last condition is a source of activator at the back of the bud, $I(x, y) = \exp(-x^2 - (y + 10.5)^2) + \exp(-x^2 - (y - 22.5)^2) + \exp\left(-(x - 15)^2 - (y - 22.5)^2\right)$.
